# Lifestyle, Oxidative Stress, and Antioxidants: Back and Forth in the Pathophysiology of Chronic Diseases

**DOI:** 10.3389/fphys.2020.00694

**Published:** 2020-07-02

**Authors:** Mehdi Sharifi-Rad, Nanjangud V. Anil Kumar, Paolo Zucca, Elena Maria Varoni, Luciana Dini, Elisa Panzarini, Jovana Rajkovic, Patrick Valere Tsouh Fokou, Elena Azzini, Ilaria Peluso, Abhay Prakash Mishra, Manisha Nigam, Youssef El Rayess, Marc El Beyrouthy, Letizia Polito, Marcello Iriti, Natália Martins, Miquel Martorell, Anca Oana Docea, William N. Setzer, Daniela Calina, William C. Cho, Javad Sharifi-Rad

**Affiliations:** ^1^Department of Medical Parasitology, Faculty of Medicine, Kerman University of Medical Sciences, Kerman, Iran; ^2^Department of Chemistry, Manipal Institute of Technology, Manipal Academy of Higher Education, Manipal, India; ^3^Department of Biomedical Sciences, University of Cagliari, Cagliari, Italy; ^4^Department of Biomedical, Surgical and Dental Sciences, Milan State University, Milan, Italy; ^5^Department of Biological and Environmental Sciences and Technologies (Di.S.Te.B.A.), University of Salento, Lecce, Italy; ^6^Medical Faculty, Institute of Pharmacology, Clinical Pharmacology and Toxicology, University of Belgrade, Belgrade, Serbia; ^7^Department of Biochemistry, Faculty of Science, University of Bamenda, Bambili, Cameroon; ^8^CREA – Research Centre for Food and Nutrition, Rome, Italy; ^9^Department of Pharmaceutical Chemistry, H.N.B. Garhwal (A Central) University, Srinagar, India; ^10^Department of Biochemistry, Hemvati Nandan Bahuguna Garhwal University (A Central University), Srinagar, India; ^11^Department of Agriculture and Food Engineering, School of Engineering, Holy Spirit University of Kaslik, Jounieh, Lebanon; ^12^General Pathology Section, Department of Experimental, Diagnostic and Specialty Medicine – DIMES, Bologna, Italy; ^13^Department of Agricultural and Environmental Sciences, Milan State University, Milan, Italy; ^14^Faculty of Medicine, University of Porto, Porto, Portugal; ^15^Institute for Research and Innovation in Health (i3S), University of Porto, Porto, Portugal; ^16^Department of Nutrition and Dietetics, Faculty of Pharmacy, University of Concepcion, Concepcion, Chile; ^17^Unidad de Desarrollo Tecnológico, Universidad de Concepción UDT, Concepcion, Chile; ^18^Department of Clinical Pharmacy, University of Medicine and Pharmacy of Craiova, Craiova, Romania; ^19^Department of Chemistry, The University of Alabama in Huntsville, Huntsville, AL, United States; ^20^Department of Clinical Pharmacy, University of Medicine and Pharmacy of Craiova, Craiova, Romania; ^21^Department of Clinical Oncology, Queen Elizabeth Hospital, Hong Kong, China; ^22^Phytochemistry Research Center, Shahid Beheshti University of Medical Sciences, Tehran, Iran

**Keywords:** reactive oxygen species, oxidative stress, natural antioxidants, neurological disorders, cardiovascular diseases, cancer, aging, antioxidant defense

## Abstract

Oxidative stress plays an essential role in the pathogenesis of chronic diseases such as cardiovascular diseases, diabetes, neurodegenerative diseases, and cancer. Long term exposure to increased levels of pro-oxidant factors can cause structural defects at a mitochondrial DNA level, as well as functional alteration of several enzymes and cellular structures leading to aberrations in gene expression. The modern lifestyle associated with processed food, exposure to a wide range of chemicals and lack of exercise plays an important role in oxidative stress induction. However, the use of medicinal plants with antioxidant properties has been exploited for their ability to treat or prevent several human pathologies in which oxidative stress seems to be one of the causes. In this review we discuss the diseases in which oxidative stress is one of the triggers and the plant-derived antioxidant compounds with their mechanisms of antioxidant defenses that can help in the prevention of these diseases. Finally, both the beneficial and detrimental effects of antioxidant molecules that are used to reduce oxidative stress in several human conditions are discussed.

## Introduction

Many natural biological processes in our bodies, such as breathing, digesting food, metabolize alcohol and drugs, and turning fats into energy produce harmful compounds called free radicals. Free radicals are usually destroyed by our body’s natural antioxidant system. If this system an not cope properly, free radicals can trigger a negative chain reaction in the body, a reaction that can destroy the cell membrane, block the action of major enzymes, prevent cellular processes necessary for proper functioning of the body, prevent normal cell division, destroy deoxyribonucleic acid (DNA), and block energy generation (Kurutas, 2015).

Oxidative stress is reported to associate with the development of several metabolic, chronic disorders or cancers (Finkel and Holbrook, 2000; Reuter et al., 2010; Aminjan et al., 2019).

The theory of free radicals of oxygen has been known for over 50 years, however, it is only in the last two decades that their role in the development of diseases were discovered and, thus, the beneficial effects of antioxidants have been widely studied (Liu, 2019).

Free radicals play an essential role in several biological processes. Many of these are necessary for life, such as the intracellular destruction of bacteria by phagocytes, especially by granulocytes and macrophages. Researchers believe that free radicals are also involved in some cellular signaling processes, known as redox signaling (Finkel and Holbrook, 2000). At low-to-moderate amounts, ROS are beneficial both in regulating processes involving the maintenance of homeostasis as well as a wide variety of cellular functions (Finkel and Holbrook, 2000; Bhattacharyya et al., 2014).

Excessive ROS production determines structural modification of cellular proteins and the alteration of their functions, leading to cellular dysfunction and disruption of vital cellular processes (Finkel and Holbrook, 2000; Kaminski et al., 2002). High ROS levels cause lipid, protein, and DNA damage. In particular, ROS can break the lipid membrane and increase membrane fluidity and permeability. Protein damage involves site-specific amino acid modification, peptide chain fragmentation, cross-linked reaction products aggregation, electric charge alteration, enzymatic inactivation, and proteolysis susceptibility (Ayala et al., 2014). Finally, ROS can damage DNA through oxidizing deoxyribose, breaking strand, removing nucleotides, modifying bases and crosslinking DNA-protein (Sharma et al., 2012; Cadet and Wagner, 2013; Cadet et al., 2017; Liang et al., 2020).

Primary oxygen free radicals are superoxide and hydroxyl radical. They are derived from molecular oxygen under chemical reduction conditions. Excessive amounts of these free radicals can lead to cell damage and apoptosis, contributing to many diseases such as cancer, stroke (Tsatsakis A. et al., 2019), myocardial infarction, diabetes, and other significant conditions (Padureanu et al., 2019). Many cancers are thought to be the result of interactions between free radicals and DNA that lead to mutations that affect the cell cycle and which then leads to neoplasia (Reuter et al., 2010).

Because free radicals are necessary for life, the body has several enzymatic mechanisms to minimize radically induced damage and to protect against excessive production of free radicals. Antioxidants play a vital role in these defense mechanisms. In healthy organisms, protection against the harmful effects of reactive oxygen species is achieved by maintaining a delicate balance between oxidants and antioxidants. The continuous production of free radicals in aerobic organisms must therefore be equalized by a similar rate of antioxidant consumption. Enzymatic or non-enzymatic, antioxidants are substances that prevent the formation of free radicals, and seek and neutralize or repair the damage caused by them (Clark et al., 1985). The protection against oxidative damage and chronic diseases is achieved through a variety of endogenous and exogenous antioxidants (Cadet et al., 2012).

ROS homeostasis is ensured by various antioxidant systems present both in plants (Sharma et al., 2012) and the human body (Birben et al., 2012). Natural ROS production through the mitochondrial respiratory chain is involved since ROS can be metabolically beneficial, but, at the same time, harmful to cells in some conditions (Hsu et al., 2000; Poli et al., 2004; Valko et al., 2006).

Conversely, in pathological or stress conditions, ROS overwhelms antioxidant systems leading to an imbalance, which, in turn, causes oxidative stress and irreversible changes in cell compounds, including proteins, carbohydrates and lipids, in addition to being able to disrupt normal cellular-signaling mechanisms (Birben et al., 2012; Zal et al., 2014; Salehi et al., 2018; Sharifi-Rad et al., 2018).

In autoimmune diseases, free radicals can change the expression of self-antigen-type proteins, increasing their immune response or changing their antigenic profile. The immune response can also be influenced by external antioxidants such as allergens in susceptible individuals. Pollen from some plant species has been shown to contain nicotinamide adenine dinucleotide phosphate oxidase (NADPH oxidase), which induces an inflammatory response in the airways with specific symptoms due to infiltration with proinflammatory cytokines, TNF-alpha and interleukins from epithelial cells. The appearance and accumulation of intracellular prooxidant factors has the long-term effect of altering the immune response by altering the structure and, implicitly, the function of proteins or enzymes such as: interferon−gamma (IFN−γ), cluster of differentiation antigen 14 (CD14), and tumor necrosis factor-a (TNF-α) (Tsoukalas et al., 2019c).

In cancers, alteration of purine or pyrimidine in the structure of cellular DNA, which is associated with a number of other reactions that produce oxides and free radicals, may be the cause of neoplasms. If the intracellular mechanisms of repair of oxidative defects are insufficient or disturbed in turn by the oxidative factors present, there are definitive consequences in some genes or products resulting from the expression of these genes, which causes mutagenesis and modification of the apoptotic mechanism of the cell, thus resulting in the tumor cell (Buj and Aird, 2018).

In the long term, the changes spread and self-sustain with the permanent activation of the autoimmune response and the accumulation of local proinflammatory factors, for example: TNF-alpha, proteases, kinases. These factors favor tissue necrosis and accelerate tissue growth with the appearance of new modified cells that maintain the immune response and propagate the initial genetic defects with chaotic and extensive multiplication; also, oxidative stress produces structural changes of cell membranes with decreased adhesion, and the migration of altered tumor cells in neighboring tissues or in distant blood and lymph (Forni et al., 2019).

In cellular aging, two theories on the mechanisms of cellular aging are currently accepted: the mitochondrial theory and the free radical theory. They support the hypothesis that mitochondria are affected by an increased level of intracellular free radicals, which leads to the alteration of their function and a decreased cellular regenerative capacity. At the same time, the progressive accumulation of intracellular oxidizing factors that exceed the antioxidant capacity is also accepted. Under these conditions, the biological decline of the respective tissue and the reduction of the adaptive c pacity to stress appear. Subsequently, regardless of the mechanism involved, in mitochondrial DNA damage or in the direct involvement of prooxidant factors in cellular mechanisms, the cellular response to stress will produce an overexpression of proinflammatory genes with increasing levels of prooxidant factors (Liguori et al., 2018).

Oxidative stress stimulates the immune response and causes allergic diseases, such as asthma, allergic rhinitis, atopic dermatitis, or food allergies. This means that the antioxidant protection system of patients with allergic diseases is outdated compared to that of healthy individuals (Sackesen et al., 2008). Supplementation with antioxidants could therefore compensate for the increased inflammatory and oxidative stress processes in asthma patients. However, Murr et al. (2005) have shown that too much antioxidant supplementation can increase the susceptibility to allergic diseases and thus asthma, by decreasing the Th1-type immune response and increasing the Th2-type response with immunoglobulin synthesis.

The modern lifestyle associated with an unhealthy diet, lack of physical exercise, exposure to a combination of chemicals from different sources pesticides (Tsatsakis A.M. et al., 2019), heavy metals, food additives, and environmental pollution can influence the appearance of oxidative stress. It can contribute to the increasing burden of chronic diseases, as is suggested by several experimental and human studies (Fenga et al., 2017; Docea et al., 2018; Fountoucidou et al., 2019; Kostoff et al., 2020). This comprehensive review aims to provide strong evidence that antioxidants may contribute to the amelioration of some chronic-degenerative conditions, in addition to being able to promote healthy aging.

## Chronic Diseases Influenced by ROS-Modalities of Action

### The ROS Sources

**FIGURE 1:**
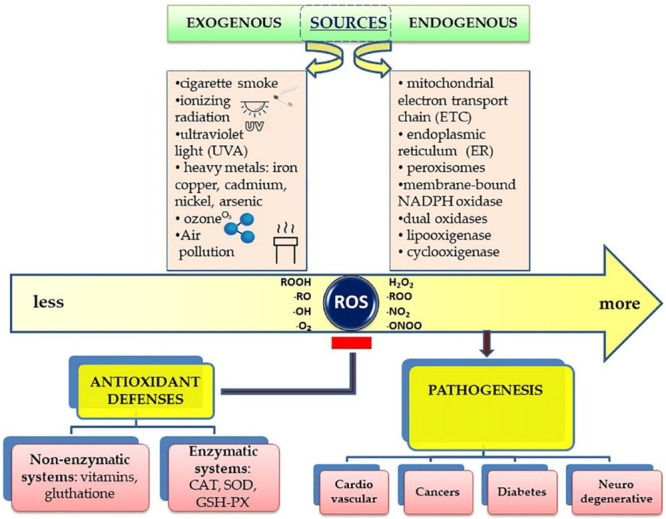
Schematic presentation of the sources of free radicals and their effects on the human body.

Free radicals are generally produced as a result of the influence of external factors, such as pollution, cigarette smoke, or internally, as a result of intracellular metabolism if the antioxidant mechanisms are overwhelmed (Figure 1).

#### Exogenous ROS

Environmental triggers, such as exposure to cigarette smoke, UV radiation, heavy metal ions, ozone, allergens, drugs or toxins, pollutants, pesticides, or insecticides, may all contribute to the increase of ROS production in cells (Antunes dos Santos et al., 2018; Mahajan et al., 2018; Oke et al., 2019).

Ionizing radiation acts by converting hydroxyl radicals, superoxides and organic radicals into organic hydroperoxides and hydrogen peroxide. Subsequently, the peroxides react with the metal ions of Fe and Cu at the cellular level through redox reactions with secondary oxidative activity. Several studies have shown that the exposure of fibroblasts to alpha particles has led to an intracellular increase of oxygen and an accelerated production of peroxide at this level (Spitz et al., 2004; Spitz and Hauer-Jensen, 2014).

Ultraviolet radiation (UVA) triggers oxidative reactions by stimulating riboflavin, porphyrins and NADPH-oxidase, with the production of 8-oxo-guanine as the main result and the decrease of intracellular glutathione (GSH) level with a return to normal after cessation of exposure (Marchitti et al., 2011).

Heavy metals play an essential role in the production of free radicals (Ściskalska et al., 2014). Iron, copper, cadmium, nickel, arsenic, and lead can induce free radicals by Fenton or Haber-Weiss type reactions, but also by direct reactions between metal ions and cellular compounds with similar effects – for example, the production of thiol type radicals. Lead triggers lipid peroxidation and increases glutathione peroxidase concentration in brain tissue. Arsenic induces the production of peroxides, superoxides, nitric oxide and inhibits antioxidant enzymes such as glutathione-transferase, glutathione-peroxidase, and glutathione-reductase by binding to the sulfhydryl group. The free radicals generated from these reactions can affect DNA, with substitutions of some DNA bases such as guanine with cytosine, guanine with thymine and cytosine with thymine (Jan et al., 2015). Exposure to ozone can affect lung function even in healthy individuals by increasing inflammatory infiltrate in the respiratory epithelium (Wu X. et al., 2019).

#### Endogenous ROS Production

The main endogenous sites of cellular redox-reactive species generation-including ROS and reactive nitrogen species (RNS) comprise mitochondrial electron transport chain (ETC), endoplasmic reticulum (ER), peroxisomes, membrane-bound NADPH oxidase (NOX) isoforms 1–5, dual oxidases (Duox) 1 and 2 complexes, and nitric oxide synthases isoforms 1–5 (NOS1–3). The complexes I and III of mitochondrial ETC produces superoxide anion (Rodriguez and Redman, 2005).

The mitochondrial ETC is considered to be the primary endogenous source of ROS but other internal sources are also present. Other sources of ROS, primarily H_2_O_2_, are microsomes and peroxisomes. Immune cells, such as macrophages and neutrophils, can also generate ROS due to their oxygen-dependent mechanisms to fight against invading microorganisms based on NOX2 isoform (Curi et al., 2016). Furthermore, dysregulated ROS signaling may contribute to a multitude of diseases associated with oxidative stress (Finkel, 2011).

ROS are produced in mitochondria during aerobic metabolism (Rodriguez and Redman, 2005). ROS generation within mitochondria (oxidative metabolism) is closely associated with ATP synthesis (oxidative phosphorylation). In aerobic organisms, the coupling of these reactions is the primary source of energy (Papa et al., 2012).

Mitochondria serve as a major ROS generator and, at the same time, as a ROS receptor. Covalent and enzymatic changes in proteins during or after protein biosynthesis as well as during protein cleavage or degradation promote disease through oxidative damage and mitochondrial dysfunction. These post-translational changes participate in the regulation of mitochondrial function through free radical species and other messengers (Hu and Ren, 2016).

Since oxidative phosphorylation is a leaky process, 0.2–5% of the electrons circulate through ETC in each round of ATP production. This produces an incompletely O_2_ reduction (Hamanaka et al., 2013).

Superoxide radicals are produced by NADPH oxidases (NOX) and, to a minor extent, as by-products of a wide number of metabolic enzymes such as cyclooxygenase (COX) 1/2, lipoxygenase, xanthine oxidoreductase (XOR) and cytochrome p450 (Finkel, 2003).

Because of the anionic properties of superoxide radicals, they diffuse through biological lipid membranes at the meager extent. They are sequentially reduced inside cells to form hydrogen peroxide and hydroxyl radical (Bartosz, 2009). Furthermore, peroxyl and alkoxyl radicals, as well as hypochlorite ions, are also formed (Valko et al., 2007).

All these types of ROS can be very harmful to cells; in fact, they can oxidize and subsequently inactivate several functions of cell components and even DNA (Valko et al., 2007). All these processes may trigger irreversible apoptotic and necrotic cell death.

Several studies indicate that human cells can also actively trigger ROS production at small doses, as part of signaling pathways, regulating cell survival and proliferation, as a defense mechanism against invaders (Bartosz, 2009; Sena and Chandel, 2012). In particular, specific enzymatic systems, such as the NOX family, dedicated explicitly to superoxide radical production with physiological signaling purposes, are developed by cells (Bedard and Krause, 2007).

In normal situations, electrons are transferred through mitochondrial ETC for oxygen reduction to water, but approximately 1–3% of electrons escape from this system and produce superoxide (Ramsay, 2019). Beyond this, other internally generated sources of ROS are present in humans, including:

(i) oxidative burst from phagocytes (white blood cells) during bacteria and virus killing and foreign proteins denaturation;

(ii) xanthine oxidoreductase (XOR) metabolism;

(ii) arachidonate pathways;

(iii) peroxisomes metabolism;

(iv) detoxification of toxic substances (i.e., vigorous exercise, chronic inflammation, and infections) (Birben et al., 2012).

ROS decrease phosphatase activity, by inhibiting catalytic regions susceptible to oxidation, and, thus, enhance protein tyrosine phosphatase (PTP) phosphorylation and influences signal transduction (Bedard and Krause, 2007). ROS can also improve signal transduction pathways that disturb the nuclear factor-κB (NF-κB) activation and translocation of this into the nucleus. The DNA binding potential of oxidized NF-κB is significantly reduced. However, NF-κB may be decreased by TR or redox factor 1 (Kabe et al., 2005). The above provokes ROS and RNS so it can strongly affect NF-κB-dependent inflammatory signals. Cyclopentenones are electrophilic anti-inflammatory prostaglandins which are conjugated with the reactive thiols of ROS-modified peptides and proteins and thus dampens ROS-mediated NF-κB signaling (Homem de Bittencourt and Curi, 2001). On the other hand, endogenous stress has an intracellular origin. Several studies have highlighted the role of cultural cell conditions, altering gene expression patterns of different genes and their DNA stability. Metabolic processes trigger different types of ROS, that are able to, if present at inadequate levels, oxidize DNA and induce various damage, such as double-stranded DNA breaks and deficiencies, often found in human tumors (De Bont and van Larebeke, 2004). Moreover, there are non-enzymatic reactions, like the mitochondrial respiratory chain which involves NADPH oxidase, XOR, uncoupled endothelial NOS, cytochrome P450 enzymes, lipoxygenase and COX (Sena and Chandel, 2012; Battelli et al., 2014a).

Cellular oxidative metabolism produces free radicals and organic peroxides as by-products during cellular mitochondrial electron transport or through metal-catalyzed oxidation of metabolites and oxidoreductases (Forman and Torres, 2002; Hussain et al., 2016).

Moreover, nitric oxide is produced in hypoxic conditions in a respiratory chain reaction, and RNS may trigger reactive species production, such as reactive aldehydes, malondialdehyde (MDA) and 4-hydroxy-2-non-enal (Hussain et al., 2016). ROS can alter the cell’s redox status and, thus, send a signal. However, an imbalance in this protective mechanism can lead to damage in cell molecules, such as DNA, proteins and lipids, resulting in cell death by necrotic and apoptotic processes (Bhattacharyya et al., 2014; Hussain et al., 2016). Stimulated ROS production was first described in phagocytic cells, including neutrophils and macrophages, during phagocytosis or stimulation with a wide variety of agents through NADPH oxidase activation. This was named “the respiratory burst” due to transient oxygen consumption (Lamy et al., 1996; Peake and Suzuki, 2004). The respiratory burst of neutrophils, as well as their degranulation, constitute a defensive response to host tissue damage, whether induced by mechanical (muscle damage during exercise, thermal stress), chemical or infectious stimuli (Lamy et al., 1996; Peake and Suzuki, 2004).

Nowadays, ROS production has also been observed in a variety of cells other than phagocytes, and their implication in physiologic signaling is well documented (Di Meo et al., 2016).

#### The Role of Lifestyle in Oxidative Stress Response

Lifestyle: smoking, alcohol consumption, adequate or inappropriate diet, exercise, training or untrained condition, contribute to oxidative stress. Some research has shown the presence of reactive oxygen species and muscle level and their role in regulating muscle activity. Skeletal muscle fibers continuously generate reactive oxygen species at a low level, which increases during muscle contraction. They exert multiple direct and indirect effects on muscle activity (contractility, excitability, metabolism, and calcium homeostasis) and are involved in skeletal muscle fatigue during strenuous exercise (Pingitore et al., 2015).

Exhausting exercises, long exercises, overtraining syndrome, and overcoming limits as a phase of the initial onset of overtraining syndrome, induce a significant response to oxidative stress. Instead, moderate exercise, low intensity training, and prolonged training, improve endogenous antioxidant status. Reactive oxygen species play an important role in cell signaling and in regulating the expression of antioxidant genes. Physical exertion produces a hyperregulation of the nuclear factor kappa B and mitogen-activated protein kinase that activates gene expression of a number of enzymes and proteins with an important role in maintaining oxidative/antioxidant intracellular homeostasis (Antonioni et al., 2019).

Physical exercise is considered the main treatment of non-pharmacological therapies along with lifestyle changes for various chronic diseases, especially cardiovascular diseases (Ren and Taegtmeyer, 2015). The results of some experimental studies have highlighted the role of autophagy, a conservative process of catabolism for the degradation and recycling of cellular organs and nutrients, in the cardiovascular benefits offered by training (Wu N. N. et al., 2019). Regular exercise as a unique form of physiological stress is able to trigger adaptation, while autophagy, especially selective mitochondrial autophagy, also called mitophagy, allows for such cardiovascular adaptation (Wu N. N. et al., 2019).

Cigarette smoke comprises a series of oxidants, free radicals, as well as organic components (e.g., nitric oxide and nitric superoxide) where the lung level activates the accumulation of neutrophils and macrophages, which increases the production of oxidants locally (Valavanidis et al., 2009).

### Biochemical/Molecular Targets and Chronic Diseases Mechanistically Linked to ROS

Endogenous ROS comprises the by-products of cellular metabolism in aerobic organisms.

At low concentrations, they are usually involved in different cell processes, such as proliferation, differentiation, and apoptosis, like a second messenger in cell signaling (Salehi et al., 2018). ROS production within cells under physiological condition is dependent on mitochondria respiration, NOX, uncoupled NOS and XOR.

The increase in ROS levels, its production in inappropriate cellular compartments or its production with defective forms during oxidative processes can trigger the development of numerous chronic-degenerative disorders, leading to severe damage to bio macromolecules (Chen et al., 2012; Gandhi and Abramov, 2012; Salehi et al., 2018). Oxidative stress, as a result of the imbalance between oxidative and antioxidative processes in cells, therefore plays an essential role in the pathogenesis of numerous chronic-degenerative disorders.

#### ROS and Cardiovascular Diseases

The main cardiovascular risk factors, such as hypertension and hypercholesterolemia contribute to enhancing ROS generation, leading to oxidative stress (Li et al., 2014). From all these cardiovascular risk factors, hypertension is an essential factor in the development of cardiovascular diseases (CVD) (Elahi et al., 2009).

ROS has a dual role in cardiovascular physiopathology.

Small amounts of ROS in the cardiovascular system could provide remarkable benefits: anti-atherosclerotic, pro-angiogenesis and endogenous cardioprotective effects (Taverne et al., 2013). Large numbers of ROS induce the loss of cell viability, since oxidative stress is involved in the development and/or progression of CVD, such as endothelial dysfunction, atherosclerosis, myocardial ischemia/reperfusion damage, heart failure, arrhythmias (Taverne et al., 2013; Sharifi-Rad J. et al., 2020).

In CVD, gene expression is altered due to oxidative stress. Increased ROS levels modulate transcription factor activity, especially NF-κB, activator protein-1 (AP-1) and the peroxisome proliferators-activated receptor (PPAR) family of transcriptional activators (Elahi et al., 2009).

As a result of increasing ROS generation, one of the first events in atherogenesis, as well as in other CVDs correlated with endothelial dysfunction, is the oxidative modification of low-density lipoprotein (LDL) (Singh et al., 2002; Perrotta and Aquila, 2015). Indeed, both cell membranes and LDL, enriched with phospholipids, are highly sensitive to oxidative modification. Oxidized phospholipids, through receptor-mediated or receptor-independent pathways, can therefore then activate endothelial cells, induce endothelium adhesion molecules expression, attract monocytes, have endothelium cytotoxic effects, and increase proinflammatory gene activity and cellular growth factors (Esper et al., 2006). All of these processes provoke endothelial dysfunction, platelet aggregation, and metalloproteinase expression and favor thrombogenesis (Esper et al., 2006).

In atherosclerotic plaque, increased matrix metalloproteinase expression and activity triggered by oxidative stress lead to its rupture and consequent thrombosis (He and Zuo, 2015). The NF-κB activity in atherosclerosis is mainly due to oxidized LDL (Singh et al., 2002). At the same time, upregulated NF-κB is detected in smooth muscle cells, endothelial cells, macrophages and T cells of atherosclerotic plaques (Mach et al., 1998).

In the blood vessel wall, all layers can produce ROS under pathological conditions, and most of them are primarily derived from NOX (Reid, 2001). Due to increased ROS levels, NO bioavailability is decreased, and consequently, endothelium-dependent relaxation is reduced (Chen J.Y. et al., 2018).

Cardiac myocytes have a more significant number of mitochondria than other cells and use higher oxygen levels for energy production in the form of ATP. In myocytes, ROS trigger cardiac injury, both oxidizing essential proteins for excitation-contraction and decreasing NO bioactivity (Hare and Stamler, 2005).

In myocardial ischemia, mitochondrial electron transport is imbalanced, leading to ATP depletion, acidosis, mitochondrial depolarization, intracellular Ca^2+^ overload and, then, apoptosis (Wattanapitayakul and Bauer, 2001). Furthermore, oxidative stress produced in mitochondria induces mitochondrial DNA (mtDNA) damage and leads to CVD. In myocardial ischemia, hypoxia and reoxygenation trigger an increase in free radical production in cardiac tissue (Elahi et al., 2009). ROS produced during reoxygenation cause direct oxidative damage to cellular components and lead to indirect damage through the activation of localized inflammation (Gutteridge and Halliwell, 2000). In heart failure, excessive ROS production is based on increased activity of XOR and NOX (Battelli et al., 2014b). Increased ROS production is a consequence of prolonged endoplasmic reticulum stress and mitochondrial-derived oxidative stress in cardio-metabolic disorders.

The function of these organelles is correlated with Ca^2+^ release and uptake and, as a result of oxidative stress, abnormal Ca^2+^ handling may cause arrhythmias. Furthermore, some disturbance in these organelles activates signaling pathways that alter cardiac ion channels function or expression, involved in the generation of an action potential that promotes arrhythmogenesis (Tse et al., 2016).

The administration of cytostatics to humans is followed by cardiotoxicity due to increased plasma levels of ROS and lipid peroxidation products and decreased plasma and tissue levels of antioxidants. Myocardial changes that occur after treatment include: myocyte loss through apoptosis or necrosis, loss of myofibrils, distension of the sarcoplasmic reticulum, and mitochondrial ballooning. Recent studies on transgenic mice have shown that in cardiotoxicity induced by Doxorubicin, free radicals can be counteracted by metallothionein and liensinine (Kang, 1996; Liang et al., 2020).

#### ROS and Cancers

Cancer development in humans is a complex process that includes cellular and molecular changes mediated by various endogenous and exogenous stimuli (Docea et al., 2012; Sani et al., 2017; Mishra et al., 2018). It has been established that oxidative DNA damage is one of the key characteristics of carcinogenesis (Smith et al., 2016). Cancer initiation and promotion are associated with chromosomal defects and activation of oncogenes by free radicals (Glasauer and Chandel, 2014). A common form of injury is the formation of hydroxylated DNA bases, considered an important event in chemical carcinogenesis. They interfere with healthy cell growth by causing genetic mutations and altering normal gene transcription. Oxidative lesions also produce many changes in the structure of DNA (Li et al., 2015).

ROS involvement in a different stage of carcinogenesis has been shown in various model systems. Excessive amounts of these free radicals can lead to cell damage and apoptosis. Many forms of cancer are considered to be the result of free radicals and DNA reactions, leading to mutations that can affect the cell cycle and lead to neoplasia (Pizzino et al., 2014, 2017).

ROS overproduction has an impact on cancer cell proliferation, metastatic potential, and it is associated with invasiveness and poor prognosis (Liou et al., 2016). ROS contributes to cancer cell migration through various mechanisms: (i) matrix degradation, (ii) cell-cell contact, (iii) cytoskeleton remodeling, regulation of gene expression, (iv) invadopodia formation (Pizzino et al., 2014).

For example, mitochondria-derived ROS has an impact on initial extracellular matrix contact, NOX-derived ROS are involved in invadopodia formation. At the same time, ROS increase in cytosol plays a significant role in cytoskeleton remodeling (Herrera et al., 2004). The effect of ROS on cancers depends on the type of organ, as well as on the grade of disease progression.

Skin carcinogenesis and exposure to UVA: the ultraviolet component A sunlight (UV-A with the wavelength 320–400 nm) has the potential to generate oxidative stress in cells and tissues, so that endogenous and exogenous antioxidants strongly influence the biological effects of UVA (Sage et al., 2012). The physiological doses of UVA determine the expression of some genes (collagenase, hem oxygenase-1, and nuclear oncogenes), whose effects can be significantly increased by removing intracellular GSH or by increasing the lifetime of molecular oxygen. Repeated exposure of human skin to UV radiation leads not only to skin carcinogenesis but also to photo-aging through DNA damage (Cortat et al., 2013; Saha et al., 2017).

Hydroxyl radicals can bind to DNA and produce 8-OH deoxyguanosine (8-OHdG), which consequently increases the risk of mutation. 8-OHdG can also initiate cancer-inducing mutagenesis by transforming GC pairs to TA pairs during DNA replication (Sova et al., 2010). Therefore, 8-OHdG molecules may be used as indicators for free radicals’ detection during DNA mutagenesis (Forcados et al., 2017). Additionally, increased cancer cell proliferation requires high ATP levels that lead to ROS accumulation, particularly at initial stages of cancer genesis.

In cancer cells, there is the condition of constant oxidative stress induced by mitochondrial dysfunction and metabolic changes. In fact, under normal circumstances, increased ROS levels stimulate cell death, but cancer cells overcome that by activating numerous oncogenes, which then induce nuclear factor erythroid 2-related factor 2 (NRF2) expression. NRF2 is the primary regulator of cell survival that raises cancer progression by protecting cancer cells from ROS and DNA damage (Jaramillo and Zhang, 2013).

ROS are implicated in cancer progression, promoting cyclin D1 expression, extracellular signal-regulated kinase (ERK) and JUN N-terminal kinase (JNK) phosphorylation, and MAPK activation (Saha et al., 2017). However, cancer cells enable proliferation, avoiding ROS-induced apoptosis, despite high mutagenesis.

In neoplastic disorders, ROS promote protein oxidation and lipid peroxidation. Moreover, ROS trigger toxic protein carbonyls formation which has a significant impact on other proteins or lipids (Benfeitas et al., 2017). In addition, as a result of lipid peroxidation, cancer cells accumulate products, such as 4-hydroxy-2-non-enal, one of the most studied products of phospholipid peroxidation, owing to its reactivity and cytotoxicity.

#### ROS and Neurodegenerative Disorders

In the brain, not all neuronal groups are equally sensitive to oxidative stress. For instance, neurons with longer axons and multiple synapses require more energy for axonal transport or long-term plasticity (Salehi et al., 2019c; Tsatsakis A. et al., 2019). High ATP demand, in combination with dysfunctional mitochondria, make these neuron groups more sensitive to degeneration (Wang and Michaelis, 2010). Correctly, dopaminergic neurons are exposed to additional oxidative stress produced by the dopamine metabolism, generating H_2_O_2_ and dopamine autoxidation, which generates superoxide (Delcambre et al., 2016; Buga et al., 2019).

Alzheimer’s, Parkinson’s, Huntington’s, amyotrophic lateral sclerosis and Friedreich’s ataxia comprise the most common neurodegenerative disorders (Reddy, 2009; Nussbaum et al., 2017; Salehi et al., 2020a). During aging, mutations in mtDNA accumulate, cytosolic calcium dysregulates, and ETC function decreases, making aging one of the major risk factors contributing to neurodegeneration (Payne and Chinnery, 2015). The oxidized molecules of DNA, proteins and lipids found in the brain tissue of post-mortem patients with neurodegenerative disorders highlight the role of oxidative stress in these diseases (Sharifi-Rad M. et al., 2020). Another cause of neurodegenerative diseases is a defective use of metals by the brain, by the intervention of mutant proteins, formed as a result of oxidative stress (Niedzielska et al., 2016). In the case of Alzheimer disease, a protein called amyloid beta (Aβ), consisting of 40 amino acid residues, is present in all the cells of the body, under normal, harmless and even beneficial conditions, as it is a natural antioxidant (Danielson and Andersen, 2008; Li et al., 2013). It has been found that in modified protein formations Aβ plaques, which are formed in the case of Alzheimer’s disease outside the affected neurons, in areas that control cognitive functions, quantities of three, up to four times higher than normal copper, zinc and iron are fixed (Riederer et al., 1989).

One explanation is the accumulation in the brain of a modified form of the Ab protein (consisting of 42 amino acid residues), which fails to properly bind metals, promotes oxidative processes; by reacting in self-defense, neurons produce antioxidants in increased quantities, including the modified form of the Aβ protein, which thus becomes an antioxidant pro-oxidant, amplifying oxidative disasters by initiating chain reactions (Danielson and Andersen, 2008).

Mutations of the superoxide dismutase 1 (SOD1) protein have been linked to another neurodegenerative disease that affects motility (familial amyotrophic lateral sclerosis) (Huai and Zhang, 2019).

In its unmodified form, SOD1 is a natural antioxidant that prevents the formation of peroxide anion as a dangerous reactive form of oxygen (Saccon et al., 2013).

The mutant forms of this protein fixate a much smaller amount of metals than the usual form, which results in the formation of an excess of peroxynitrite (ONOO^–^) affecting the motor neurons required for normal functioning, causing severe motor disorders (Pasinelli et al., 2004).

The excessive use of glucose for energy production makes the brain especially susceptible to oxidative stress, and mitochondrial ETC is the primary ROS source (Cobley et al., 2018). Most of the ROS present in the brain derive from mitochondrial ETC complex I and III (ETC I and III), as O_2_^–^ by-products (Andreyev et al., 2005).

Monoamine oxidase (MAO) is also a great source of ROS, especially in Parkinson’s. Indeed, the main targets for mitochondria-generated ROS are mitochondrial permeability transition pore (MPTP), poly (ADP-ribose) polymerase (PARP), and mtDNA (Gandhi and Abramov, 2012).

Other oxidant sources arise from NADPH oxidase, present in astrocytes, microglia and neurons, while NOS inhibition has shown neuroprotective effects (Abramov et al., 2005).

In the pathogenesis of neurodegeneration, many processes are included, such as protein misfolding and aggregation, abnormal kinase-signaling pathways, neuronal calcium dysregulation, and even impaired synaptic transmission (Gandhi and Abramov, 2012). Detached abnormally aggregated proteins that are morphologically characteristic of many neurodegenerative diseases in neurons and/or glial cells, as well as the rupture of metal ions homeostasis, are highly associated with oxidative stress (Chen et al., 2012). Mechanisms of action of ROS: these affect proteins by modifying them in oxidative forms, which tend to form aggregates (Blokhuis et al., 2013). Protein aggregates then inhibit proteasomes, the main organelles in the cell for degradation of abnormal proteins (Chen et al., 2012; Takalo et al., 2013). Accumulation of modified proteins with an inability to be destroyed in the proteasome stimulate more ROS formation and form a vicious cycle, a phenomenon included in neurodegenerative diseases related to oxidative stress (Chen et al., 2012; Takalo et al., 2013).

#### ROS, Diabetes, and Metabolic Syndrome

Many metabolic contexts can lead to conditions of oxidative stress. A condition in which oxidation is an important pathogenetic link is type 2 diabetes. In this disease, insulin resistance is the basic component, to which a compensatory hypersecretion of insulin is linked. Reactive oxygen species can induce inactivation of signaling mechanisms between insulin receptors and the glucose transport system, leading to insulin resistance (Chen X.F. et al., 2018).

On the other hand, diabetes itself is a generator of oxidative stress, with atherogenetic consequences. Hyperglycemia induces the generation of superoxide ions in endothelial cells at the mitochondrial level. In diabetes, electron transfer and oxidative phosphorylation are decoupled, resulting in the production of superoxide anions and inefficient ATP synthesis. Therefore, preventing the damage caused by oxidation is a therapeutic strategy in diabetes. Increased levels of free fatty acids with consecutive accumulation of intramyocellular lipids were thought to be the cause of insulin resistance and beta-pancreatic cell death.

Studies have shown that both glucose and free fatty acids can initiate the formation of free radicals through mitochondrial mechanisms and NADPH oxidase in muscles, adipocytes, beta cells and other cell types. Free fatty acids penetrate cellular organs, including mitochondria, where high levels of reactive oxygen species can cause peroxidation and damage. Recent studies show that type II diabetes and insulin resistance are associated with a decrease in mitochondrial oxidative function in skeletal muscle. Moreover, in this type of diabetes, the mitochondria are smaller, rounder and more likely to produce superoxide. Disorders of the mitochondrial transport chain, excessive generation of reactive species and lipoperoxides, as well as decreases in antioxidant mechanisms have also been observed in diabetes and obesity.

Diabetes has a number of complications over time, of which macrovasculopathy is very important. The increase in cardiovascular risk in patients with diabetes can be explained by the association between diabetes hypertension, dyslipidemia and coronary atherosclerotic disease. However, other mechanisms are also involved, such as the effects of hyperglycemia on endothelial function, the effects of glucose and fatty acids on myocardial cells, at the structural level but also of gene expression (Aroor et al., 2012; Karam et al., 2017).

Diabetic cardiovascular complications are caused by impaired cardiac microvascular function. In addition to the structural and functional changes that occur in diabetic cardiomyopathy, other mechanisms can be targeted pharmacologically. Sodium-glucose co-transporter-2 (SGLT2) inhibitors are the first class of antidiabetic drugs that have reduced the risk of heart failure in type 2 diabetes (Karam et al., 2017). Empagliflozin has an indication to reduce cardiovascular mortality in patients with diabetes and atherosclerotic disease. A recent study demonstrated the beneficial effect of empagliflozin on cardiac microvascular injury in diabetes and the protective mechanism against oxidative stress in mitochondria (Zhou et al., 2018).

Another recent study showed that aminoguanidine has a beneficial effect on diabetes-induced heart abnormalities. Aminoguanidine saves contractile abnormalities and diabetes-induced cardiac remodeling. This was explained by inhibition of endoplasmic reticulum stress and induction of autophagy (Pei et al., 2018).

Insulin resistance, abdominal obesity, atherogenic dyslipidemia, endothelial dysfunction, high blood pressure, hypercoagulability, genetic predisposition and chronic stress are the main factors underlying the metabolic syndrome. Metabolic syndrome is often characterized by oxidative stress, a condition in which there is an imbalance between the production and inactivation of reactive oxygen species. Increased generation of reactive oxygen species, decreased activity of antioxidant systems or both mechanisms may be involved in the occurrence of oxidative stress (Karam et al., 2017).

A study showed that lenalidomide attenuates oxidative cardiovascular tissue damage and apoptosis in obese mice by inhibiting tumor necrosis factor (Zhu et al., 2014).

#### ROS and Aging

Postulated over 60 years ago, the “free radical theory of aging” pointed to ROS as a cause of damage accumulation in cell constituents and connective tissues. This accumulation of losses in cells would be the reason for aging and aging-associated degenerative diseases (Tsoukalas et al., 2019a). Aging can be caused by both genetic and external factors, such as incorrect diet, improper physical exercise, chronic drug use, untreated inflammatory conditions, smoking, and alcohol abuse.

Today, while there are several theories of aging, the basic principle of most of them is still oxidative stress (Finkel and Holbrook, 2000; Payne and Chinnery, 2015). The major systems involved in overproduction of oxidative stress in cells are mitochondria and NOX (Bedard and Krause, 2007). NOX comprises a family of several membrane-associated enzymes, located in mitochondria of many cell types, and it has been shown in different studies that its enhanced activity and/or expression is included in age-associated diseases (Zhang et al., 2004; Park et al., 2008; Egea et al., 2017).

In the aging process, it has been noticed that high-molecular protein aggregates accumulate in cells (Davalli et al., 2016). Predominantly, these aggregates are made from proteins, with the remainder consisting of various lipids (Barrera, 2012; Takalo et al., 2013; Tsoukalas et al., 2019b). Most of the proteins aggregated are oxidized/modified by different reactive metabolites, and they could bind to cellular proteins. Thus, the crucial point for protein homeostasis maintenance is the degradation of these aggregates.

The central place for cell damaged protein degradation is the proteasome, which recognizes only unfolded proteins as degradation targets (Saez and Vilchez, 2014). Proteasome inhibition prevents further degradation of newly formed oxidized proteins and increases protein aggregation formation in cells (Takalo et al., 2013; Saez and Vilchez, 2014). Besides that, proteasome becomes dysfunctional during aging.

While proteasomal dysfunction is correlated with age progression and protein aggregation, proteasome activation slows the aging progress down and increases longevity (Chondrogianni et al., 2014). In many invertebrate models and cell lines, it has been shown that the overexpression of different proteasomal regulatory or catalytic subunits or treatment with specific compounds has positive effects on proteasome activity (Saez and Vilchez, 2014).

Along with the “free radical theory of aging” popularization, this has started the trend of antioxidant supplement consumption (Conti et al., 2016). Recently, most of the data have indicated that antioxidant supplementation does not decrease the incidence of age-related diseases (Schottker et al., 2015).

## Antioxidant Defenses

**FIGURE 2:**
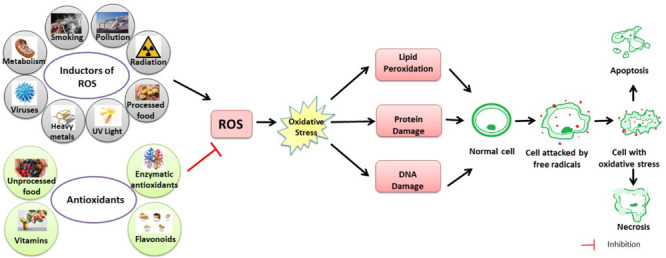
Schematic figure of the link between ROS, oxidative stress and their effects on the human body. Oxidative stress is the imbalance that occurs when there is an increased production of free radicals that exceeds the body’s ability to neutralize it. Alteration of chemical reactions at the cellular level leads to the appearance of free radicals and peroxides that affect the intracellular structures – proteins, lipids, DNA, with the disruption of intrinsic mechanisms at this level. Free radicals are normally produced in the body due to the influence of external factors, such as pollution, cigarette smoke, or internal, due to intracellular metabolism when antioxidant mechanisms are exceeded.

Antioxidants break radical chain reactions, preventing oxidative stress-related damage (Da Pozzo et al., 2018; Figure 2).

Their role requires acting both in hydrophilic and hydrophobic cellular environments, so their chemical structure is quite heterogeneous.

There are enzymatic and non-enzymatic antioxidants (Banafsheh and Sirous, 2016), as shown in Figure 1. but, from a nutritional perspective, a more informative classification can be made between endogenous and exogenous classes.

**FIGURE 3:**
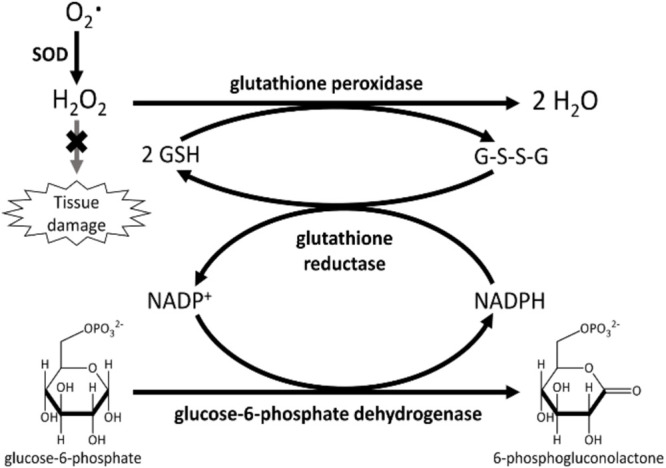
Primary enzymes (SOD or peroxidases) act directly in scavenging ROS. Secondary enzymes, such as glutathione reductase and glucose-6-phosphate dehydrogenase, support the action of primary enzymes regenerating NAPDH and reduced glutathione.

The first class comprises all antioxidants that cells can synthesize from smaller building blocks. Accordingly, all enzymatic antioxidants are endogenous, as well as some non-enzymatic ones (i.e., thiols antioxidants and coenzyme Q10, Figure 3).

On the contrary, exogenous antioxidants have to be ingested through the diet, since their synthesis is impossible in eukaryotic cells. So, particular attention should be paid on this latter class, since this is the most unpredictable component in cellular redox balance.

Antioxidants can be divided into two categories depending on their solubility: water soluble and liposoluble (Lazzarino et al., 2019).

Water soluble antioxidants are best absorbed in the body because the vegetables and fruits that contain such antioxidants, also contain water. On the other hand, they are rapidly eliminated from the body through the urine. Water-soluble antioxidants include polyphenols, but also vitamin C (Lazzarino et al., 2019).

Liposoluble antioxidants, fat-soluble antioxidants are those that are absorbed in the presence of fats. Therefore, in the absence of fats, the body cannot absorb and use these antioxidants. It is important to note, however, that they are not easily removed from the body and can accumulate over time, exceeding the healthy level. Vitamin E is an example of a fat-soluble antioxidant (Lazzarino et al., 2019).

### Enzymatic Antioxidants

Several enzymes obstruct free radicals’ formation, some of them act directly in scavenging ROS (primary enzymes), whereas “secondary enzymes” play an indirect role by supporting other endogenous antioxidants (Banafsheh and Sirous, 2016). This is the case, for instance, for glucose-6-phosphate dehydrogenase that regenerates NADPH, essential for primary enzyme action (Figure 2).

#### Primary Enzymes

Primary enzymes act directly on the main ROS arising from incomplete O_2_ reduction, O_2_^–^ and H_2_O_2_. SOD scavenges the former, whereas CAT and GPX remove the latter. SOD (E.C. 1.15.1.1) is a metalloenzyme, catalyzing superoxide anion dismutation to H_2_O_2_ and molecular oxygen, as shown in reaction 1 (Singh et al., 2017). In turn, H_2_O_2_ can be removed by the other enzymatic antioxidant systems.

(1) $$2O_{2}^{-}\cdot +2H^{+}\overset{S\,O\,D}{\to }H_{2}O_{2}+O_{2}$$

SODs can be divided into four groups, with different metal cofactors. Copper-zinc SOD is most abundant in chloroplasts, cytosol and extracellular space. Iron SOD is found in plant cytosol and in microbial cells, whereas manganese SODs are mitochondrial (Perera et al., 2017). SOD also competes for superoxide anion with NO. Therefore, SOD also indirectly reduces the formation of another deleterious ROS, peroxynitrite (ONOO^–^, reaction 2), and increases the NO biological availability, an essential modulator for endothelial function.

(2) $$O_{2}^{-}\cdot +NO\cdot \to ONOO^{-}$$

CAT (E.C. 1.11.1.6) is a tetrameric ferriheme oxidoreductase, which catalyzes H_2_O_2_ dismutation to water and gaseous oxygen, as shown in reaction 3 (Grigoras, 2017).

(3) $$2\,H_{2}\,O_{2}\,\overset{C\,A\,T}{\to }2\,H_{2}\,O+O_{2}$$

CAT is mainly located in peroxisomes, and despite being ubiquitous, the highest activity is present in liver and red blood cells. CAT works with a two-step mechanism, somewhat resembling the formation in the first step of a peroxidase-like compound I intermediate, CpdI (reaction 4) (Alfonso-Prieto et al., 2009; Zucca et al., 2014), which in turn decomposes to resting-state upon reaction with a second hydrogen peroxide molecule (reaction 5):

(4) $$Catalase\left(Fe^{3+}\right)+H_{2}O_{2}\to CpdI^{+}\left(Fe^{4+}=0\right)+H_{2}O$$

(5) $$CpdI^{+}\left(Fe^{4+}=0\right)+H_{2}O_{2}\to Catalase\left(Fe^{3+}\right)+H_{2}O+O_{2}$$

A NADPH molecule is bound to each subunit, minimizing H_2_O_2_–mediated inactivation [105]. CAT is one of the enzymes with the highest known *k*_cat_(more than 10^6^ s^–1^) in all known proteins, close to a diffusion-controlled reaction (Tovmasyan et al., 2015). The role of CAT versus peroxynitrite has been much debated, but recent advances suggested CAT’s ability to scavenge ONOO^–^ (Gebicka and Didik, 2009).

GPX (E.C. 1.11.1.19) is a selenium-dependent oxidoreductase, which uses H_2_O_2_ or organic hydroperoxide as the oxidant, and the tripeptide GSH as the electron donor (Cardoso et al., 2017), in a typical class I peroxidase catalytic cycle (reactions 6 and 7).

(6) $$H_{2}\,O_{2}+2\,G\,S\,H\,\overset{G\,P\,x}{\to }2\,H_{2}\,O+G\,S-S\,G$$

(7) $$R\,O\,O\,H+2\,G\,S\,H\,\overset{G\,P\,x}{\to }R\,O\,H+H_{2}\,O+G\,S-S\,G$$

The GPX family is composed of eight isoenzymes (GPX1-8). Each enzyme presents peculiar features. GPX1, 2, 3, and 4 incorporate selenocysteine (a non-standard amino acid, where the sulfur atom of cysteine is replaced by selenium). GPX6 contains selenium only in humans, but not in rodents; whereas GPX5, 7, and 8 do not have selenium, but a “normal” cysteine instead (Cardoso et al., 2017).

During the catalytic cycle, selenocysteine is converted from selenol (Enz-SeH) to selenenic acid (Enz-SeOH), with concomitant reduction of H_2_O_2_ or ROOH. Then, the first GSH molecules yield selenenyl sulfide intermediate (Enz-Se-SG). An incoming second GSH molecule attacks Enz-Se-SG, regenerating the enzymatic resting form Enz-SeH, releasing the oxidized and dimerized GSSG (Cardoso et al., 2017).

Another important class of enzymatic peroxide scavenger is PRDX. Six different classes of PRDX have been identified (Poole and Nelson, 2016), showing either one (1-Cys PRDX) or two (2-Cys PRDX) redox-active cysteine residues (Park et al., 2016). The PRDX catalytic cycle involves H_2_O_2_ decomposition and the subsequent regeneration of the resting enzyme, using a small cysteine protein thioredoxin (Trx) as the reductant (reactions 8 and 9). Trx shows two vicinal cysteines (in the typical CXXC motif), forming, in turn, a disulfide internal bridge upon oxidation. In the case of PRDX6 isoform, Trx can be replaced by GSH.

(8) $$R\,D\,X_{r\,e\,d\,u\,c\,e\,d}+H_{2}\,O_{2}\to P\,R\,D\,X_{o\,x\,i\,d\,i\,z\,e\,d}+2\,H_{2}\,O$$

(9) $$P\,R\,D\,X_{o\,x\,i\,d\,i\,z\,e\,d}+T\,r\,x_{r\,e\,d\,u\,c\,e\,d}\to P\,R\,D\,X_{r\,e\,d\,u\,c\,e\,d}+T\,r\,x_{o\,x\,i\,d\,i\,z\,e\,d}$$

#### Secondary Enzymes

All the enzymatic activities described above rely on the continuous regeneration of the reduced form of reductants (mainly GSH and Trx). This is usually performed by some reductases, NADPH-dependent (such as glutathione reductase E.C. 1.8.1.7 and thioredoxin reductase E.C. 1.8.1.9). However, as shown in Figure 2, reduced NADPH is, in turn, needed by these reductases for their continuous action. So, enzymes responsible for the constant NADPH production can be considered secondary antioxidants, as their misfunction could affect the whole ROS balance. The main NADPH metabolic source is the pentose phosphate pathway, through the first two enzymatic activities: glucose-6-phosphate dehydrogenase (E.C. 1.1.1.49) and 6-phosphogluconate dehydrogenase (E.C. 1.1.1.44). However, other contributions come from the malic enzyme (E.C. 1.1.1.40) and a recently identified folate-dependent mechanism (Fan et al., 2014).

### Non-enzymatic Antioxidants

Some chemical molecules of low-molecular-weight can also directly act as antioxidants. In this case, their action is not catalytic, always needing antioxidant regeneration or its supply from the diet. Non-enzymatic antioxidants can therefore be divided into endogenous (if the eukaryotic cell is able to synthesize it) and exogenous (if the antioxidant needs to be ingested mandatorily through the diet).

#### Endogenous Non-enzymatic Antioxidants

Both GSH and Trx can act also act potent ROS scavengers (including ⋅OH, H_2_O_2_, organic peroxides, and ONOO^–^) directly, without enzymatic help.

**FIGURE 4:**
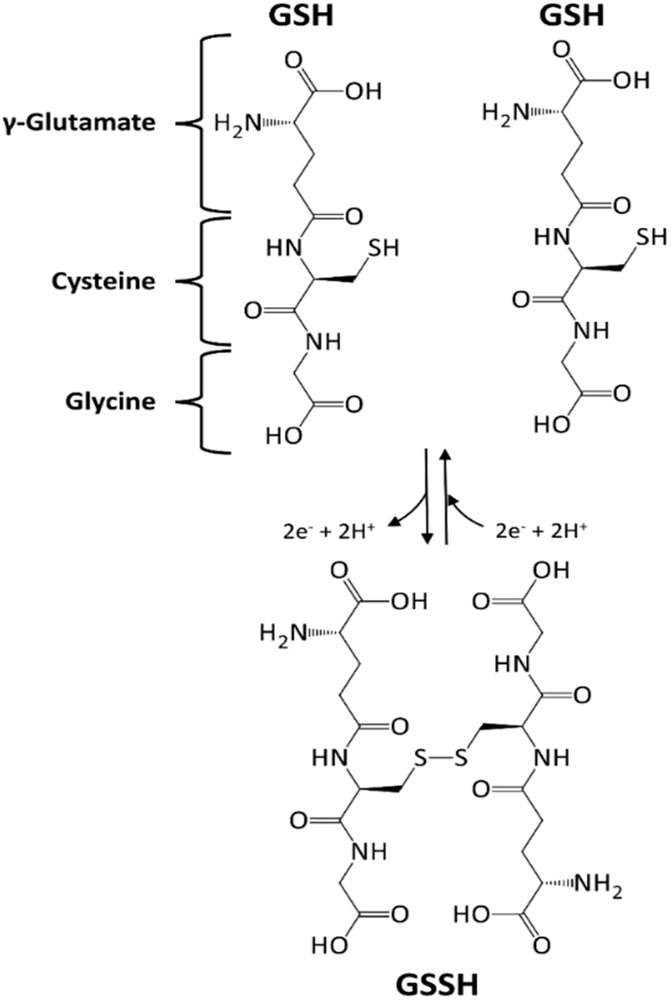
Glutathione (GSH), a tripeptide with an active –SH function. GSH undergoes a redox cycle, dimerizing with a disulfide bridge formation.

GSH (γ-glutamyl-cysteinyl-glycine, Figure 4) is a tripeptide, mainly distributed in cytosol, but also in nuclei, peroxisomes and mitochondria. Despite being ubiquitous, the liver is the leading site for its synthesis (Banafsheh and Sirous, 2016). GSH biosynthesis is an endergonic process (ATP hydrolysis is coupled), in which firstly glutamate and cysteine condense to yield γ-glutamylcysteine (reaction catalyzed by glutamate-cysteine ligase, E.C. 6.3.2.2). This unusual γ-peptidic bond protects it from the common peptidases action. In the final step, GSH synthetase (E.C. 6.3.2.3) adds a glycine residue to the α-amino group of cysteine (Hasanuzzaman et al., 2017).

α-Lipoic acid (1,2-dithiolane-3-pentanoic acid, Figure 4) is a disulfide compound that undergoes a redox cycle similar to GSH. Accordingly, it scavenges reactive ROS, and regenerate vitamins C and E, and GSH in their active forms (Kucukgoncu et al., 2017). Lipoic acid also has a role in metal chelation, preventing Fenton-like radical reactions (Zhang and McCullough, 2016). Nevertheless, even small proteins, such as Trx and glutaredoxin can similarly function as thiol antioxidants, showing redox-active mono- or di-cysteine motif (CXXC). Both proteins can be in turn reduced back to their active form, directly by GSH or indirectly by NADPH (Banafsheh and Sirous, 2016).

**FIGURE 5:**
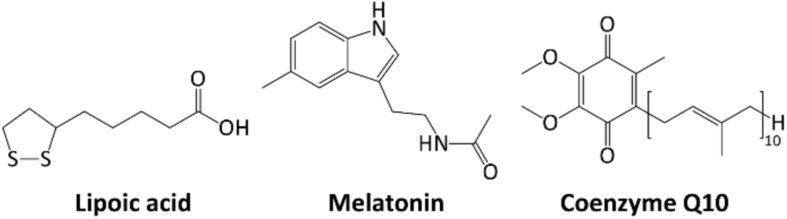
Chemical structures of Lipoic acid, Melatonin, Coenzyme Q10.

Melatonin (*N*-acetyl-5-methoxytryptamine, Figure 5) is a neurohormone derived from amino acid tryptophan. It is involved in circadian rhythms but also acts as a potent antioxidant, protecting cell membranes against lipid peroxidation (Beyer et al., 1998).

It has been described to be more effective in ROS scavenging than vitamin E, GSH, vitamin C and β-carotene (Watson, 1998).

Coenzyme Q10 or ubiquinone (2,3-dimethoxy-5-methyl-6-polyisoprene parabenzoquinone, Figure 5) is an isoprenoid antioxidant present in cell membranes, essential for ETC (Tafazoli, 2017). Its synthesis starts from oligomerization of isoprenoid building blocks, isopentenyl pyrophosphate and dimethylallyl pyrophosphate (both arising from the mevalonate pathway and the key enzyme 3-hydroxy-3-methyl-glutaryl-CoA reductase (E.C. 1.1.1.88). The resulting decaprenyl diphosphate is then conjugated with a tyrosine derivative to yield the active form of the coenzyme. It is one of the few liposoluble antioxidants, ensuring lipoproteins and lipids protection from radical chain reactions, peroxidation and oxidative damage (Lee et al., 2017). In its active form (quinol), coenzyme Q10 can scavenge several ROS or regenerate other oxidized antioxidants (including vitamins C and E). In turn, the quinone form can be reduced back by several NAD(P)H-dependent enzymatic systems.

#### Exogenous Non-enzymatic Antioxidants

Exogenous antioxidants need to be supplemented continuously through the diet since their synthetic pathways are usually present only in microbial or plant cells. Vitamins, two of which show prominent antioxidant effects, such as vitamins C and E, belong to essential class of molecules.

Vitamin C (ascorbic acid) exists in two redox forms: ascorbic acid (AA) is the reduced form, which is deprotonated at physiological pH (thus, occurring in its anion form, ascorbate). Due to its high electron-donating power, AA can undergo two-electron oxidation, yielding dehydroascorbic acid (DHA). One-electron oxidation of AA is also possible, generating a semi-dehydro-ascorbyl radical (Kocot et al., 2017). DHA can be regenerated to the active AA form by GSH- or Trx-dependent mechanisms. Humans do not express the enzyme L-gulonolactone oxidase (E.C. 1.1.3.8), avoiding the possibility of endogenous synthesis. Thus, AA must be ingested by food (or supplements), particularly tomatoes, pineapples, watermelons and all citrus fruits (Banafsheh and Sirous, 2016). AA effectively quenches ROS, both directly and cooperatively regenerating oxidized vitamin E, GSH, and carotenoids. Scavenged ROS can be superoxide anion, ^1^O_2_, H_2_O_2_, organic peroxides, ●OH or hypochlorous acid (HClO).

**FIGURE 6:**
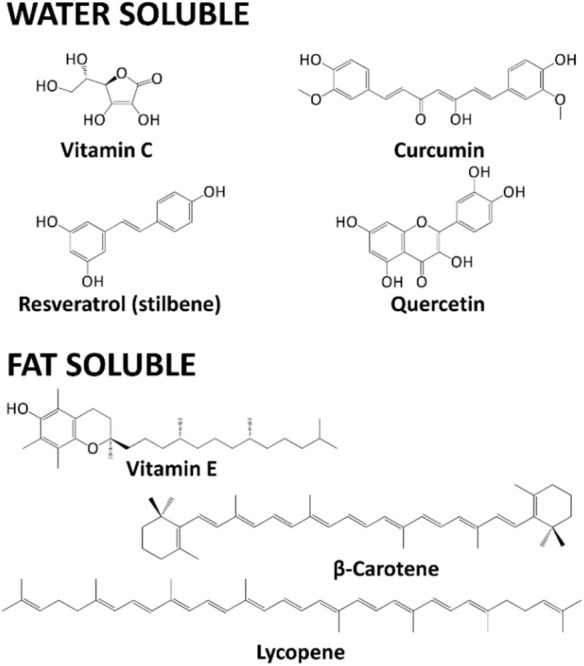
Chemical structures of Vitamin C, Curcumin, Resveratrol, Quercetin, Vitamin E, β-carotene, Lycopene.

Vitamin E is a fat-soluble vitamin, mostly found in several vegetable oils, nuts, broccoli and fish. Eight different forms have been reported (α-, β-, γ-, and δ-tocopherol, and α-, β-, γ-, and δ-tocotrienol), but α-tocopherol has the highest antioxidant activity, especially in cell membranes (Salehi et al., 2020b). A variously methyl-substituted chromanol ring characterizes tocopherols. A long phytyl chain gives the hydrophobicity (Figure 6).

On the contrary, tocotrienols bear an unsaturated isoprenoid chain. α-Tocopherol is able to undergo hydrogen transfer to several ROS, including ^1^O_2_, superoxide anion and peroxyl radicals. The oxidized and radical derivative of vitamin E is then reduced by the AA.

Carotenoids are a broad class of tetraterpenes, widely distributed among plants. Carotenoids are divided into unoxygenated “carotenes.” On the contrary, if oxygen atoms are present, “xanthophylls” is the correct term (Meybodi et al., 2017). Carotenes are also vitamin A precursors. Carotenoids protect plant chlorophyll, acting as accessory pigments during photosynthesis. Thus, they are intensely colored (red, orange, or yellow) molecules.

Carotenoids have been suggested to be chemopreventive agents in cancer (Marti et al., 2016; Salehi et al., 2019a), even if the real effect is still debated (Panic et al., 2017). Their biological activities also include ROS scavenging (Hernández-Almanza et al., 2016; Salehi et al., 2019b). β-Carotene comprises one of the most diffused carotenes, being the primary pro-vitamin A precursor, and it is found mainly in carrots, pumpkins, mangoes and apricots. Lycopene is another well-known acyclic carotene, not being a precursor of vitamin A, and is found primarily in tomatoes and other red fruits, but not in strawberries and cherries. Indeed, carotenoids are strong ROS scavengers, operating a very particular physical and chemical ^1^O_2_ quenching (Banafsheh and Sirous, 2016). In the physical mechanism, the carotenoid electron-rich structure absorbs ^1^O_2_ excess energy, reaching an excited state. The conjugated double bond structure in carotenoids is responsible for this ability. The excited state then decays to the ground state, losing the surplus energy as heat. During this cycle, the structure of this molecule stays unchanged.

Polyphenols are a large class of plant secondary metabolites, whose synthesis is usually possible only in these organisms (Sanjust et al., 2008; Salehi et al., 2019a). The key enzyme [phenylalanine ammonia-lyase (PAL), EC 4.3.1.5] is only present in plants (Rasouli et al., 2016). PAL catalyzes the non-oxidative deamination of phenylalanine to *trans*-cinnamic acid, which is the fundamental building block for polyphenol synthesis in the phenylpropanoid pathway (Ertani et al., 2016). Several biological functions have been ascribed to polyphenols, including anti-inflammatory, antioxidant, antimicrobial and antimelanogenesis effects (Zucca et al., 2016; Barbieri et al., 2017; Da Pozzo et al., 2018), suggesting their use in the human diet and as food supplements. The most widely recognized polyphenols’ classes are flavonoids, phenolic acids, stilbenes (including resveratrol), tannins, coumarins, curcuminoids and lignans. For instance, one of the most studied polyphenols has been curcumin, gaining a lot of attention also for nutraceutical applications. This phytochemical has, in fact, potent activity as a scavenger for superoxide anions, H_2_O_2_, lipid peroxides, ●OH, and several RNS (Imam et al., 2017). Curcumin can also increase GSH cellular levels (Banafsheh and Sirous, 2016).

Epigallocatechin-3-gallate (EGCG) is a well-known antioxidant. The green tea catechins include catechin, epicatechin, epigallocatechin, epicatechin gallate, and epigallocatechin gallate (Barbieri et al., 2017). Catechins can react with superoxide anions and ●OH, and is also able to chelate metal ions (Imam et al., 2017; Roychoudhury et al., 2017).

Flavonoids, in addition to its strong antioxidant properties, quench ROS formation inhibiting several enzymes and chelating metals involved in radical chain reactions (Banafsheh and Sirous, 2016). In fact, flavonoids present shallow redox potentials, allowing them to directly reduce highly reactive ROS with high redox potentials, such as superoxide anion, peroxyl radicals and ●OH by hydrogen atom transfer mechanism (HAT) (Salehi et al., 2019d). Furthermore, flavonoids can also affect free metal ion concentrations. In the cell environment, these can promote ROS formation reacting with H_2_O_2_ and generating highly reactive ●OH, in a series of Fenton and Fenton-like reactions. Indeed, flavonoids have the well-known capacity to chelate several metal ions (such as iron and copper), blocking free radical generation (Kumar and Pandey, 2013). For instance, quercetin is one of the most diffused flavonols present in broccoli, apples, grapes, onions and soybeans, with both iron-chelating and iron-stabilizing abilities (Kumar and Pandey, 2013). On the other hand, catechol and galloyl-derivatives are generally well-known metal chelators (Jomova and Valko, 2011). So, they can all exert their antioxidant activity by blocking Fenton-like reactions.

Organosulfur compounds have also been suggested as potent antioxidants. The most studied are probably some sulfur-containing metabolites present in garlic (mainly *S*-allyl-mercapto cysteine, *S*-allyl cysteine, and diallyl sulfide, diallyl trisulfide) (Kimura et al., 2017). These organosulfur are also responsible for typical garlic flavor. Their antioxidant actions include scavenging ROS and inhibiting lipids peroxidation (Borek, 2001; Miltonprabu et al., 2017).

Several minerals, in small amounts, are also essential for some enzymatic antioxidant activities. They are therefore sometimes regarded as antioxidants themselves. For instance, selenium is a necessary component of GPX (Battin and Brumaghim, 2009), while copper, zinc, and manganese are fundamental for SOD activity.

## Pro-Oxidative Role of Antioxidants: Double-Sided Sword

The balance between ROS production and purification maintains homeostasis of the body, but is most often directed to the formation of free radicals and involvement in the pathophysiology of chronic diseases. The use of antioxidant supplements containing multivitamins and minerals has always grown in popularity among consumers. But some recent studies have not shown any beneficial effect of antioxidant therapy. Oxidative stress has a dual character: it is both harmful and beneficial to the body, because some ROS are signaling molecules on cellular signaling pathways. Lowering the level of oxidative stress through antioxidant supplements is therefore not beneficial in such cases (Ye et al., 2004; Ray et al., 2012).

Antioxidants are also prone to oxidation since oxidation and reduction reactions do not happen in isolation. AA, a potent antioxidant, mediates several physiological responses. Still, it can also act as a pro-oxidant when it combines with iron and copper, reducing Fe^3+^ to Fe^2+^ (or Cu^2+^ to Cu^+^), which in turn reduces H_2_O_2_ to hydroxyl radicals (Duarte and Lunec, 2005). This reaction is responsible for oxidative stress-produced DNA damage. However, the role of AA as anti- or pro-oxidant depends on the dose used, as observed in the case of ischemia-induced oxidative stress (Seo and Lee, 2002).

On the other hand, the pro-oxidant/antioxidant activity of β-carotene and lycopene has been reported to be dependent on its interaction with biological membranes and other co-antioxidant molecules, like vitamins C or E (Young and Lowe, 2001). With increased oxygen tension, carotenoids tend to lose their antioxidant potential. Otherwise, α-tocopherol, a powerful antioxidant, becomes pro-oxidant at high concentrations (Cillard and Cillard, 1980). Interestingly, when it reacts with a free radical, it becomes a radical in itself. If there is not enough AA for its regeneration, it will remain in that highly reactive state (Lü et al., 2010).

Flavonoids can also act as pro-oxidants depending on the concentrations used (Prochazkova et al., 2011). Indeed, dietary phenolics may act as pro-oxidants in the presence of O_2_ and transition metals, like iron and copper, catalyzing phenolics’ redox cycling and leading to ROS and phenoxyl radicals’ formation, with consequent damage in DNA, lipids and other biological components (Galati and O’Brien, 2004). Nevertheless, the extent to which these phytochemicals are capable of acting as anti- or pro-oxidants *in vivo* is still poorly understood, and this topic undoubtedly requires further research.

## Clinical Studies Related to Efficacy of Antioxidants in Prevention of Chronic Diseases

The hypothesis that antioxidants could protect against cancer because they can neutralize reactive oxygen species (ROS) that can damage DNA has long been issued. In laboratory and animal studies, the presence of elevated levels of exogenous antioxidants has been shown to prevent the types of free radicals that have been associated with the development of cancer.

A few randomized studies evaluating the role of antioxidant supplements for cancer prevention were conducted in collaboration with the National Cancer Institute (Goodman et al., 2011). No data were obtained to justify that they are effective in primary cancer prevention. An analysis in the United States concluded that there is no clear scientific evidence for the benefits of vitamin and mineral supplements in cancer prevention.

It is important to point out that there have been cases where people who have resorted to these types of supplements have encountered an unfavorable evolution of the disease. Preclinical studies also report that antioxidants have contributed to the expansion of tumor processes in animal models. A well-known case is that of vitamin A, for which the administration of high doses in supplements has been associated with an increased risk of cancer. Vitamin A can be obtained preformed from animal sources or plant products, derived from β-carotene. Supplements are recommended only in special cases, at the doctor’s advice and especially in areas where there are frequent deficits (Jerome-Morais et al., 2011).

β-Carotene is an orange pigment found in fruits and vegetables (carrots, sweet potatoes, mangoes, apricots), and in the body it is converted to vitamin A. A normal intake has a beneficial effect against the risk of cancer. However, studies have shown a correlation between the administration of β-carotene supplements and the risk of bladder cancer, as well as the risk of lung cancer in smokers (Lin et al., 2009).

In another study, the administration of α-tocopherol and β-carotene for lung cancer did not change the incidence of lung cancer. However, α-tocopherol supplements have been shown to be effective in prostate cancer whose incidence is reduced (Goodman et al., 2011).

A trial evaluated the effectiveness of long-term supplementation with vitamin E and vitamin C in the risk of developing cancer. One of the findings of the study was that these types of supplements do not reduce the risk of prostate cancer or the overall risk of cancer in men of middle age or older. No significant results were obtained regarding the risk of colorectal or lung cancer (Gaziano et al., 2009).

Vitamin E and C supplements showed poor results in many studies. According to a clinical study entitled Physicians’ Health Study II conducted between 1997 and 2007, which included over 14,000 men over 50, the risk of major cardiovascular events was not reduced by supplementation with vitamins E or C (Lin et al., 2009).

Women’s Health Study (WHS) is another comprehensive study in the United States involving nearly 40,000 healthy women, with a minimum of 45 years of age who received 600 IU of vitamin E administered on alternate days, and who were followed for a period of 10 years (Lee et al., 2005). There was a reduction in cardiovascular mortality, but no significant effect was observed on overall mortality. The authors concluded that vitamin E supplementation for the prevention of cardiovascular disease among healthy women is not justified. Moreover, cancer mortality is not significantly influenced by vitamin E supplementation (Lee et al., 2005).

The Selenium and Vitamin E Cancer Prevention Trial (SELECT) which included over 35,000 men over the age of 50, showed that selenium and vitamin E supplements do not prevent prostate cancer. In addition, a study even suggsted that vitamin E supplements increase the risk of prostate cancer by 17% compared to a placebo (Klein et al., 2011).

## Discussion

This article summarizes the evidence from a large number of meta-analyzes covering the pathophysiological impact of antioxidants on the most common chronic diseases. The main criticism of the review is that the data were extracted from meta-analyzes and not from individual studies, but this can be considered an advantage because meta-analyzes provide the highest degree of evidence.

In the case of antioxidants, studies show that more does not necessarily mean better. Consuming superfoods does not compensate for other unhealthy eating habits or an unbalanced lifestyle. Free radicals, as well as antioxidants, can have beneficial effects on the body. Therefore, we are talking about a balance and not a negative role attributed to free radicals and a positive one to antioxidants.

Degradation of nucleic acids, proteins, lipids or other cellular components are among the effects that an excessive concentration of free radicals can generate. Many diseases that are intensely discussed today are associated with the influence of oxidative stress – cancer, cardiovascular disease, Alzheimer’s disease, autoimmune diseases. Risk factors leading to free radicals include air pollution, ionizing radiation, prolonged exercise, infections, excessive consumption of polyunsaturated fatty acids (Poprac et al., 2017).

On the other hand, antioxidants are considered to be the solution to these problems – substances that neutralize free radicals. In reality, the term “antioxidant” refers to a chemical property of a substance to donate electrons. In some situations, some substances act as antioxidants, in other situations they become prooxidants, depending on the chemical composition of the environment in which they are. There are many types of antioxidants, and the role in the body and the mechanisms by which they act are different. One misconception is that one antioxidant can be replaced with another, having the same effect. In fact, each has its own unique biological properties (Chen X.F. et al., 2018).

There is also a significant difference between taking antioxidants from food and administering an isolated substance as a supplement. Many substances that demonstrate beneficial effects in the laboratory do not work when introduced into the human body. Many antioxidants do not have good bioavailability. The concentration of antioxidants such as polyphenols is sometimes so low in the blood that no significant effect is observed (Fernández-García et al., 2012). Fruits and vegetables contain bioactive substances that in many cases do not work as antioxidants if we consider them outside of the body. But they work as antioxidants when they are in the body, because they activate their own antioxidant mechanisms. These bioactive substances are the secret behind vegetable consumption (Kurutas, 2015).

Antioxidant supplements may have different health benefits. On the one hand, it is possible that other substances present in food are responsible for the positive effects on health, not necessarily a certain type of antioxidant, but the synergistic effect of several substances. On the other hand, the chemical structure of antioxidants in food is often different from that identified in supplements. An example is vitamin E. There are eight variants of vitamin E in the foods we eat, while the supplements used in most studies contain only one form (Firuzi et al., 2011).

Studies also frequently include healthy people, for whom oxidative stress on the body is not significant to determine a risk of disease. Antioxidants can benefit certain categories of patients in whom there is a real, documented imbalance, but it may not bring anything extra for a person who gets a sufficient amount of nutrients from their diet.

Observational studies analyze the trends, or habits of certain large population groups. In many, all the risk factors that could influence the course of the study can be controlled, and demonstrating a cause-effect relationship is difficult. We also cannot rely on small studies, carried out over a short period of time and using very concentrated substances extracted from different plant or animal products, to say that we have a superfood. Nutrition is a complex science, and at the moment we can only rely on the evidence accumulated so far. A food rich in antioxidants will not compensate for an unhealthy lifestyle.

## Concluding Remarks and Perspectives

Oxidative stress can be reduced by approaching a balanced lifestyle. Nutrition plays a critical role, and the best treatment against oxidative stress is antioxidants. Oxidative stress plays an important role in the pathogenesis of potentially severe conditions. In the long term, increasing the level of prooxidant factors can cause structural defects in mitochondrial DNA and alterations in enzymatic functionality or cellular structures, with the appearance of functional, structural abnormalities or aberrations in gene expression. It has also been shown that in addition to metabolic products, other external agents can have a prooxidant effect, which has led to the conclusion that lifestyle and diet can play an important role in controlling oxidative stress.

Plant-derived bioactive molecules have gained pivotal attention in recent years, given their therapeutic relevance in both disease prevention and treatment, whether using the whole plants, plant extracts or even the isolated constituents with full phytochemical profiles. The daily intake of a wide variety of phytochemicals has shown to be chemopreventive. It might hold promise for add-on treatment for several diseases, including cancer, diabetes, cardiovascular disease and neurodegenerative disorders. Larger randomized trials are needed to obtain clear scientific evidence on the benefits or risks of antioxidant supplementation during cancer treatment.

Antioxidants are also prone to oxidation, and therefore their use as foods (or supplements) should be carefully considered because oxidation and reduction reactions do not happen in isolation. The intake of high doses of antioxidants has been increasingly highlighted since there is increasing evidence of some detrimental effects. The study of their chemical components as future prophylactic and therapeutic agents would be of particular interest, as they are more effective and safer than those widely available.

In conclusion, oxidative stress is an important pathogenetic link for humans and studies in this field may be important elements in the future, to better understand and manage various diseases.

## Author Contributions

JS-R and MS-R contributed to the conceptualization. NA, PZ, EV, and LD contributed to the validation investigation. EP, JR, PT, EA, IP, YE, and MB contributed to the resources. AP, MN, and AD: data curation. MS-R, AD, LP, MI, NM, MM, WS, DC, WC, and JS-R contributed to the review and editing. All authors contributed to the writing of the manuscript. All authors read and approved the final manuscript and contributed equally to the manuscript.

## Conflict of Interest

The authors declare that the research was conducted in the absence of any commercial or financial relationships that could be construed as a potential conflict of interest.

## References

[B1] Abramov A. Y. Jacobson J. Wientjes F. Hothersall J. Canevari L. Duchen M. R. (2005). Expression and modulation of an NADPH oxidase in mammalian astrocytes. *J. Neurosci.* 25 9176–9184. 10.1523/jneurosci.1632-05.2005 16207877 PMC6725750

[B2] Alfonso-Prieto M. Biarnes X. Vidossich P. Rovira C. (2009). The molecular mechanism of the catalase reaction. *J. Am. Chem. Soc.* 131 11751–11761. 10.1021/ja9018572 19653683

[B3] Aminjan H. H. Abtahi S. R. Hazrati E. Chamanara M. Jalili M. Paknejad B. (2019). Targeting of oxidative stress and inflammation through ROS/NF-kappaB pathway in phosphine-induced hepatotoxicity mitigation. *Life Sci.* 232:116607. 10.1016/j.lfs.2019.116607 31254582

[B4] Andreyev A. Y. Kushnareva Y. E. Starkov A. A. (2005). Mitochondrial metabolism of reactive oxygen species. *Biochemistry* 70 200–214. 15807660 10.1007/s10541-005-0102-7

[B5] Antonioni A. Fantini C. Dimauro I. Caporossi D. (2019). Redox homeostasis in sport: do athletes really need antioxidant support? *Res. Sports Med.* 27 147–165. 10.1080/15438627.2018.1563899 30596287

[B6] Antunes dos Santos A. Ferrer B. Marques Gonçalves F. Tsatsakis A. M. Renieri E. A. Skalny A. V. (2018). Oxidative stress in methylmercury-induced cell toxicity. *Toxics* 6:47. 10.3390/toxics6030047 30096882 PMC6161175

[B7] Aroor A. R. Mandavia C. Ren J. Sowers J. R. Pulakat L. (2012). Mitochondria and oxidative stress in the cardiorenal metabolic syndrome. *Cardiorenal Med.* 2 87–109. 10.1159/000335675 22619657 PMC3357146

[B8] Ayala A. Munoz M. F. Arguelles S. (2014). Lipid peroxidation: production, metabolism, and signaling mechanisms of malondialdehyde and 4-hydroxy-2-nonenal. *Oxid. Med. Cell. Longev.* 2014:360438. 10.1155/2014/360438 PMC4066722 24999379

[B9] Banafsheh A. A. Sirous G. (2016). Studies on oxidants and antioxidants with a brief glance at their relevance to the immune system. *Life Sci.* 146 163–173. 10.1016/j.lfs.2016.01.014 26792059

[B10] Barbieri R. Coppo E. Marchese A. Daglia M. Sobarzo-Sanchez E. Nabavi S. F. (2017). Phytochemicals for human disease: An update on plant-derived compounds antibacterial activity. *Microbiol. Res.* 196 44–68. 10.1016/j.micres.2016.12.003 28164790

[B11] Barrera G. (2012). Oxidative stress and lipid peroxidation products in cancer progression and therapy. *ISRN Oncol.* 2012 137289–137289. 23119185 10.5402/2012/137289 PMC3483701

[B12] Bartosz G. (2009). Reactive oxygen species: destroyers or messengers? *Biochem. Pharmacol.* 77 1303–1315. 10.1016/j.bcp.2008.11.009 19071092

[B13] Battelli M. G. Bolognesi A. Polito L. (2014a). Pathophysiology of circulating xanthine oxidoreductase: new emerging roles for a multi-tasking enzyme. *Biochim. Biophys. Acta* 1842 1502–1517. 10.1016/j.bbadis.2014.05.022 24882753

[B14] Battelli M. G. Polito L. Bolognesi A. (2014b). Xanthine oxidoreductase in atherosclerosis pathogenesis: not only oxidative stress. *Atherosclerosis* 237 562–567. 10.1016/j.atherosclerosis.2014.10.006 25463089

[B15] Battin E. E. Brumaghim J. L. (2009). Antioxidant activity of sulfur and selenium: a review of reactive oxygen species scavenging, glutathione peroxidase, and metal-binding antioxidant mechanisms. *Cell Biochem. Biophys.* 55 1–23. 10.1007/s12013-009-9054-7 19548119

[B16] Bedard K. Krause K. H. (2007). The NOX family of ROS-generating NADPH oxidases: physiology and pathophysiology. *Physiol. Rev* 87 245–313. 10.1152/physrev.00044.2005 17237347

[B17] Benfeitas R. Uhlen M. Nielsen J. Mardinoglu A. (2017). New challenges to study heterogeneity in cancer redox metabolism. *Front. Cell Dev. Biol.* 5:65. 10.3389/fcell.2017.00065 28744456 PMC5504267

[B18] Beyer C. E. Steketee J. D. Saphier D. (1998). Antioxidant properties of melatonin–an emerging mystery. *Biochem. Pharmacol.* 56 1265–1272. 10.1016/s0006-2952(98)00180-4 9825724

[B19] Bhattacharyya A. Chattopadhyay R. Mitra S. Crowe S. E. (2014). Oxidative stress: an essential factor in the pathogenesis of gastrointestinal mucosal diseases. *Physiol. Rev.* 94 329–354. 10.1152/physrev.00040.2012 24692350 PMC4044300

[B20] Birben E. Sahiner U. M. Sackesen C. Erzurum S. Kalayci O. (2012). Oxidative stress and antioxidant defense. *World Allergy Organ. J.* 5 9–19. 23268465 10.1097/WOX.0b013e3182439613 PMC3488923

[B21] Blokhuis A. M. Groen E. J. Koppers M. Van Den Berg L. H. Pasterkamp R. J. (2013). Protein aggregation in amyotrophic lateral sclerosis. *Acta Neuropathol.* 125 777–794. 23673820 10.1007/s00401-013-1125-6 PMC3661910

[B22] Borek C. (2001). Antioxidant health effects of aged garlic extract. *J. Nutr.* 131 1010s–1015s. 10.1093/jn/131.3.1010s 11238807

[B23] Buga A.-M. Docea A. O. Albu C. Malin R. D. Branisteanu D. E. Ianosi G. (2019). Molecular and cellular stratagem of brain metastases associated with melanoma. *Oncol. Lett.* 17 4170–4175. 30944612 10.3892/ol.2019.9933 PMC6444343

[B24] Buj R. Aird K. M. (2018). Deoxyribonucleotide triphosphate metabolism in cancer and metabolic disease. *Front. Endocrinol.* 9:177. 10.3389/fendo.2018.00177 29720963 PMC5915462

[B25] Cadet J. Davies K. J. A. Medeiros M. H. G. Di Mascio P. Wagner J. R. (2017). Formation and repair of oxidatively generated damage in cellular DNA. *Free Radic. Biol. Med.* 107 13–34. 10.1016/j.freeradbiomed.2016.12.049 28057600 PMC5457722

[B26] Cadet J. Ravanat J. L. Tavernaporro M. Menoni H. Angelov D. (2012). Oxidatively generated complex DNA damage: tandem and clustered lesions. *Cancer Lett.* 327 5–15. 10.1016/j.canlet.2012.04.005 22542631

[B27] Cadet J. Wagner J. R. (2013). DNA base damage by reactive oxygen species, oxidizing agents, and UV radiation. *Cold Spring Harb. Perspect. Biol.* 5:a012559. 10.1101/cshperspect.a012559 23378590 PMC3552502

[B28] Cardoso B. R. Hare D. J. Bush A. I. Roberts B. R. (2017). Glutathione peroxidase 4: A new player in neurodegeneration? *Mol. Psychiatry* 22 328–335. 10.1038/mp.2016.196 27777421

[B29] Chen J.-Y. Ye Z.-X. Wang X.-F. Chang J. Yang M.-W. Zhong H.-H. (2018). Nitric oxide bioavailability dysfunction involves in atherosclerosis. *Biomed. Pharmacother.* 97 423–428. 10.1016/j.biopha.2017.10.122 29091892

[B30] Chen X.-F. Wang L. Wu Y.-Z. Song S.-Y. Min H.-Y. Yang Y. (2018). Effect of puerarin in promoting fatty acid oxidation by increasing mitochondrial oxidative capacity and biogenesis in skeletal muscle in diabetic rats. *Nutr. Diabetes* 8 1–13. 29330446 10.1038/s41387-017-0009-6 PMC5851431

[B31] Chen X. Guo C. Kong J. (2012). Oxidative stress in neurodegenerative diseases. *Neural. Regen. Res.* 7 376–385. 25774178 10.3969/j.issn.1673-5374.2012.05.009 PMC4350122

[B32] Chondrogianni N. Sakellari M. Lefaki M. Papaevgeniou N. Gonos E. S. (2014). Proteasome activation delays aging in vitro and in vivo. *Free Radic. Biol. Med.* 71 303–320. 10.1016/j.freeradbiomed.2014.03.031 24681338

[B33] Cillard J. Cillard P. (1980). [Prooxidant effect of alpha-tocopherol on essential fatty acids in aqueous media]. *Ann. Nutr. Aliment.* 34 579–591. 7469263

[B34] Clark I. A. Cowden W. B. Hunt N. H. (1985). Free radical-induced pathology. *Med. Res. Revi.* 5 297–332. 10.1002/med.2610050303 3894833

[B35] Cobley J. N. Fiorello M. L. Bailey D. M. (2018). 13 reasons why the brain is susceptible to oxidative stress. *Redox Biol.* 15 490–503. 10.1016/j.redox.2018.01.008 29413961 PMC5881419

[B36] Conti V. Izzo V. Corbi G. Russomanno G. Manzo V. De Lise F. (2016). Antioxidant supplementation in the treatment of aging-associated diseases. *Front. Pharmacol.* 7:24. 10.3389/fphar.2016.00024 26903869 PMC4751263

[B37] Cortat B. Garcia C. C. M. Quinet A. Schuch A. P. De Lima-Bessa K. M. Menck C. F. M. (2013). The relative roles of DNA damage induced by UVA irradiation in human cells. *Photochem. Photobiol. Sci.* 12 1483–1495. 23824260 10.1039/c3pp50023c

[B38] Curi R. Newsholme P. Marzuca-Nassr G. N. Takahashi H. K. Hirabara S. M. Cruzat V. (2016). Regulatory principles in metabolism-then and now. *Biochem. J.* 473 1845–1857. 10.1042/bcj20160103 27354561

[B39] Da Pozzo E. De Leo M. Faraone I. Milella L. Cavallini C. Piragine E. (2018). Antioxidant and antisenescence effects of bergamot juice. *Oxid. Med. Cell. Longev.* 2018:9395804. 10.1155/2018/9395804 PMC6079356 30116497

[B40] Danielson S. R. Andersen J. K. (2008). Oxidative and nitrative protein modifications in Parkinson’s disease. *Free Radic. Biol. Med.* 44 1787–1794. 10.1016/j.freeradbiomed.2008.03.005 18395015 PMC2422863

[B41] Davalli P. Mitic T. Caporali A. Lauriola A. D’arca D. (2016). ROS, cell senescence, and novel molecular mechanisms in aging and age-related diseases. *Oxid. Med. Cell. Longev.* 2016:3565127. 10.1155/2016/3565127 PMC4877482 27247702

[B42] De Bont R. van Larebeke N. (2004). Endogenous DNA damage in humans: a review of quantitative data. *Mutagenesis* 19 169–185. 10.1093/mutage/geh025 15123782

[B43] Delcambre S. Nonnenmacher Y. Hiller K. (2016). “Dopamine metabolism and reactive oxygen species production,” in *Mitochondrial Mechanisms of Degeneration and Repair in Parkinson’s Disease*, ed. Buhlman L. (Cham: Springer).

[B44] Di Meo S. Reed T. T. Venditti P. Victor V. M. (2016). Role of ROS and RNS sources in physiological and pathological conditions. *Oxid. Med. Cell. Longev.* 2016:1245049. 10.1155/2016/1245049 PMC4960346 27478531

[B45] Docea A. O. Gofita E. Goumenou M. Calina D. Rogoveanu O. Varut M. (2018). Six months exposure to a real life mixture of 13 chemicals’ below individual NOAELs induced non monotonic sex-dependent biochemical and redox status changes in rats. *Food Chem. Toxicol.* 115 470–481. 10.1016/j.fct.2018.03.052 29621577

[B46] Docea A. O. Mitruţ P. Grigore D. Pirici D. Cãlina D. C. Gofiţã E. (2012). Immunohistochemical expression of TGF beta (TGF-β), TGF beta receptor 1 (TGFBR1), and Ki67 in intestinal variant of gastric adenocarcinomas. *Rom. J. Morphol. Embryol. Rev. Roum. Morphol. Embryol.* 53 683–692. 23188426

[B47] Duarte T. L. Lunec J. (2005). Review: when is an antioxidant not an antioxidant? A review of novel actions and reactions of vitamin C. *Free Radic. Res.* 39 671–686. 10.1080/10715760500104025 16036346

[B48] Egea J. Fabregat I. Frapart Y. M. Ghezzi P. Gorlach A. Kietzmann T. (2017). European contribution to the study of ROS: a summary of the findings and prospects for the future from the COST action BM1203 (EU-ROS). *Redox Biol.* 13 94–162. 28577489 10.1016/j.redox.2017.05.007 PMC5458069

[B49] Elahi M. M. Kong Y. X. Matata B. M. (2009). Oxidative stress as a mediator of cardiovascular disease. *Oxid. Med. Cell. Longev.* 2 259–269. 10.4161/oxim.2.5.9441 20716913 PMC2835914

[B50] Ertani A. Pizzeghello D. Francioso O. Tinti A. Nardi S. (2016). Biological activity of vegetal extracts containing phenols on plant metabolism. *Molecules* 21:205. 10.3390/molecules21020205 26867189 PMC6273273

[B51] Esper R. J. Nordaby R. A. Vilarino J. O. Paragano A. Cacharron J. L. Machado R. A. (2006). Endothelial dysfunction: a comprehensive appraisal. *Cardiovasc. Diabetol.* 5:4. 10.1186/1475-2840-5-4 PMC1434727 16504104

[B52] Fan J. Ye J. Kamphorst J. J. Shlomi T. Thompson C. B. Rabinowitz J. D. (2014). Quantitative flux analysis reveals folate-dependent NADPH production. *Nature* 510 298–302. 10.1038/nature13236 24805240 PMC4104482

[B53] Fenga C. Gangemi S. Teodoro M. Rapisarda V. Golokhvast K. Docea A. O. (2017). 8-Hydroxydeoxyguanosine as a biomarker of oxidative DNA damage in workers exposed to low-dose benzene. *Toxicol. Rep.* 4 291–295. 10.1016/j.toxrep.2017.05.008 28959652 PMC5615153

[B54] Fernández-García E. Carvajal-Lérida I. Jarén-Galán M. Garrido-Fernández J. Pérez-Gálvez A. Hornero-Méndez D. (2012). Carotenoids bioavailability from foods: from plant pigments to efficient biological activities. *Food Res. Int.* 46 438–450. 10.1016/j.foodres.2011.06.007

[B55] Finkel T. (2003). Oxidant signals and oxidative stress. *Curr. Opin. Cell Biol.* 15 247–254. 10.1016/s0955-0674(03)00002-4 12648682

[B56] Finkel T. (2011). Signal transduction by reactive oxygen species. *J. Cell Biol.* 194 7–15. 21746850 10.1083/jcb.201102095 PMC3135394

[B57] Finkel T. Holbrook N. J. (2000). Oxidants, oxidative stress and the biology of ageing. *Nature* 408 239–247. 10.1038/35041687 11089981

[B58] Firuzi O. Miri R. Tavakkoli M. Saso L. (2011). Antioxidant therapy: current status and future prospects. *Curr. Med. Chem.* 18 3871–3888. 10.2174/092986711803414368 21824100

[B59] Forcados G. E. James D. B. Sallau A. B. Muhammad A. Mabeta P. (2017). Oxidative stress and carcinogenesis: potential of phytochemicals in breast cancer therapy. *Nutr. Cancer* 69 365–374. 10.1080/01635581.2017.1267777 28103111

[B60] Forman H. J. Torres M. (2002). Reactive oxygen species and cell signaling: respiratory burst in macrophage signaling. *Am. J. Respir. Crit. Care Med.* 166 S4–S8. 12471082 10.1164/rccm.2206007

[B61] Forni C. Facchiano F. Bartoli M. Pieretti S. Facchiano A. D’arcangelo D. (2019). Beneficial role of phytochemicals on oxidative stress and age-related diseases. *BioMed Res. Int.* 2019 1–16. 10.1155/2019/8748253 31080832 PMC6475554

[B62] Fountoucidou P. Veskoukis A. S. Kerasioti E. Docea A. O. Taitzoglou I. A. Liesivuori J. (2019). A mixture of routinely encountered xenobiotics induces both redox adaptations and perturbations in blood and tissues of rats after a long-term low-dose exposure regimen: The time and dose issue. *Toxicol. Lett.* 317 24–44. 10.1016/j.toxlet.2019.09.015 31541690

[B63] Galati G. O’Brien P. J. (2004). Potential toxicity of flavonoids and other dietary phenolics: significance for their chemopreventive and anticancer properties. *Free Radic. Biol. Med.* 37 287–303. 10.1016/j.freeradbiomed.2004.04.034 15223063

[B64] Gandhi S. Abramov A. Y. (2012). Mechanism of oxidative stress in neurodegeneration. *Oxid. Med. Cell. Longev.* 2012:428010. 10.1155/2012/428010 PMC3362933 22685618

[B65] Gaziano J. M. Glynn R. J. Christen W. G. Kurth T. Belanger C. Macfadyen J. (2009). Vitamins e and c in the prevention of prostate and total cancer in men: the physicians’ health study ii randomized controlled trial. *Jama* 301 52–62. 19066368 10.1001/jama.2008.862 PMC2774210

[B66] Gebicka L. Didik J. (2009). Catalytic scavenging of peroxynitrite by catalase. *J. inorg. Biochem.* 103 1375–1379. 10.1016/j.jinorgbio.2009.07.011 19709751

[B67] Glasauer A. Chandel N. S. (2014). Targeting antioxidants for cancer therapy. *Biochem. Pharmacol.* 92 90–101. 10.1016/j.bcp.2014.07.017 25078786

[B68] Goodman M. Bostick R. M. Kucuk O. Jones D. P. (2011). Clinical trials of antioxidants as cancer prevention agents: past, present, and future. *Free Radic. Biol. Med.* 51 1068–1084. 10.1016/j.freeradbiomed.2011.05.018 21683786

[B69] Grigoras A. G. (2017). Catalase immobilization—A review. *Biochem. Eng. J.* 117 1–20. 10.1016/j.bej.2016.10.021

[B70] Gutteridge J. M. Halliwell B. (2000). Free radicals and antioxidants in the year 2000. A historical look to the future. *Ann. N.Y.Acad. Sci.* 899 136–147. 10.1111/j.1749-6632.2000.tb06182.x 10863535

[B71] Hamanaka R. B. Glasauer A. Hoover P. Yang S. Blatt H. Mullen A. R. (2013). Mitochondrial reactive oxygen species promote epidermal differentiation and hair follicle development. *Sci. Signal.* 6:ra8. 10.1126/scisignal.2003638 23386745 PMC4017376

[B72] Hare J. M. Stamler J. S. (2005). NO/redox disequilibrium in the failing heart and cardiovascular system. *J. Clin. Invest.* 115 509–517. 10.1172/jci200524459 15765132 PMC1052013

[B73] Hasanuzzaman M. Nahar K. Anee T. I. Fujita M. (2017). Glutathione in plants: biosynthesis and physiological role in environmental stress tolerance. *Physiol. Mol. Biol. Plants* 23 249–268. 10.1007/s12298-017-0422-2 28461715 PMC5391355

[B74] He F. Zuo L. (2015). Redox roles of reactive oxygen species in cardiovascular diseases. *Int. J. Mol. Sci.* 16 27770–27780. 10.3390/ijms161126059 26610475 PMC4661917

[B75] Hernández-Almanza A. Montañez J. Martínez G. Aguilar-Jiménez A. Contreras-Esquivel J. C. Aguilar C. N. (2016). Lycopene: progress in microbial production. *Trends Food Sci. Technol.* 56 142–148. 10.1016/j.tifs.2016.08.013

[B76] Herrera B. Murillo M. M. Alvarez-Barrientos A. Beltran J. Fernandez M. Fabregat I. (2004). Source of early reactive oxygen species in the apoptosis induced by transforming growth factor-beta in fetal rat hepatocytes. *Free Radic. Biol. Med.* 36 16–26. 10.1016/j.freeradbiomed.2003.09.020 14732287

[B77] Homem de Bittencourt P. I. Jr. Curi R. (2001). Antiproliferative prostaglandins and the MRP/GS-X pump role in cancer immunosuppression and insight into new strategies in cancer gene therapy. *Biochem. Pharmacol.* 62 811–819. 10.1016/s0006-2952(01)00738-9 11543717

[B78] Hsu T. C. Young M. R. Cmarik J. Colburn N. H. (2000). Activator protein 1 (AP-1)- and nuclear factor kappaB (NF-kappaB)-dependent transcriptional events in carcinogenesis. *Free Radic. Biol. Med.* 28 1338–1348. 10.1016/s0891-5849(00)00220-3 10924853

[B79] Hu N. Ren J. (2016). Reactive oxygen species regulate myocardial mitochondria through post-translational modification. *React. Oxyg. Species* 2 264–271.

[B80] Huai J. Zhang Z. (2019). Structural properties and interaction partners of familial ALS-associated SOD1 mutants. *Front. Neurol.* 10:527. 10.3389/fneur.2019.00527 31164862 PMC6536575

[B81] Hussain T. Tan B. Yin Y. Blachier F. Tossou M. C. B. Rahu N. (2016). Oxidative stress and inflammation: what polyphenols can do for us? *Oxid. Med. Cell. Longev.* 2016:7432797. 10.1155/2016/7432797 PMC5055983 27738491

[B82] Imam M. U. Zhang S. Ma J. Wang H. Wang F. (2017). Antioxidants mediate both iron homeostasis and oxidative stress. *Nutrients* 9:671. 10.3390/nu9070671 28657578 PMC5537786

[B83] Jan A. T. Azam M. Siddiqui K. Ali A. Choi I. Haq Q. M. (2015). Heavy metals and human health: mechanistic insight into toxicity and counter defense system of antioxidants. *Int. J. Mol. Sci.* 16 29592–29630. 10.3390/ijms161226183 26690422 PMC4691126

[B84] Jaramillo M. C. Zhang D. D. (2013). The emerging role of the Nrf2-Keap1 signaling pathway in cancer. *Genes Dev.* 27 2179–2191. 10.1101/gad.225680.113 24142871 PMC3814639

[B85] Jerome-Morais A. Diamond A. M. Wright M. E. (2011). Dietary supplements and human health: for better or for worse? *Mol. Nutr. Food Res.* 55 122–135. 10.1002/mnfr.201000415 21207517

[B86] Jomova K. Valko M. (2011). Advances in metal-induced oxidative stress and human disease. *Toxicology* 283 65–87. 10.1016/j.tox.2011.03.001 21414382

[B87] Kabe Y. Ando K. Hirao S. Yoshida M. Handa H. (2005). Redox regulation of NF-kappaB activation: distinct redox regulation between the cytoplasm and the nucleus. *Antioxid. Redox. Signal.* 7 395–403. 10.1089/ars.2005.7.395 15706086

[B88] Kaminski K. A. Bonda T. A. Korecki J. Musial W. J. (2002). Oxidative stress and neutrophil activation—the two keystones of ischemia/reperfusion injury. *Int. J. Cardiol.* 86 41–59. 10.1016/s0167-5273(02)00189-4 12243849

[B89] Kang Y. (1996). Chen Y, and epstein PN. Suppression of doxorubicin cardiotoxicity by overexpression of catalase in the heart of transgenic mice. *J. Biol. Chem.* 271 12610–12616. 10.1074/jbc.271.21.12610 8647872

[B90] Karam B. S. Chavez-Moreno A. Koh W. Akar J. G. Akar F. G. (2017). Oxidative stress and inflammation as central mediators of atrial fibrillation in obesity and diabetes. *Cardiovasc. Diabetol.* 16:120. 10.1186/s12933-017-0604-9 PMC5622555 28962617

[B91] Kimura S. Tung Y. C. Pan M. H. Su N. W. Lai Y. J. Cheng K. C. (2017). Black garlic: a critical review of its production, bioactivity, and application. *J. Food Drug Anal.* 25 62–70. 10.1016/j.jfda.2016.11.003 28911544 PMC9333422

[B92] Klein E. A. Thompson I. M. Jr. Tangen C. M. Crowley J. J. Lucia M. S. Goodman P. J. (2011). Vitamin E and the risk of prostate cancer: the Selenium and Vitamin E Cancer Prevention Trial (SELECT). *Jama* 306 1549–1556. 21990298 10.1001/jama.2011.1437 PMC4169010

[B93] Kocot J. Luchowska-Kocot D. Kielczykowska M. Musik I. Kurzepa J. (2017). Does vitamin C influence neurodegenerative diseases and psychiatric disorders? *Nutrients* 9:659. 10.3390/nu9070659 28654017 PMC5537779

[B94] Kostoff R. N. Heroux P. Aschner M. Tsatsakis A. (2020). Adverse health effects of 5G mobile networking technology under real-life conditions. *Toxicol. Lett.* 232 35–40. 10.1016/j.toxlet.2020.01.020 31991167

[B95] Kucukgoncu S. Zhou E. Lucas K. B. Tek C. (2017). Alpha-lipoic acid (ALA) as a supplementation for weight loss: results from a meta-analysis of randomized controlled trials. *Obes. Rev.* 18 594–601. 10.1111/obr.12528 28295905 PMC5523816

[B96] Kumar S. Pandey A. K. (2013). Chemistry and biological activities of flavonoids: an overview. *Sci. World J.* 2013:16. 10.1155/2013/162750 PMC3891543 24470791

[B97] Kurutas E. B. (2015). The importance of antioxidants which play the role in cellular response against oxidative/nitrosative stress: current state. *Nutr. J.* 15 71. 10.1186/s12937-016-0186-5 PMC4960740 27456681

[B98] Lamy M. Mathy-Hartert M. Deby-Dupont G. (1996). “Neutrophil-induced Oxidative Stress,” in *Yearbook of Intensive Care and Emergency Medicine*, ed. Vincent J. L. (Berlin: Springer), 83–95.

[B99] Lazzarino G. Listorti I. Bilotta G. Capozzolo T. Amorini A. M. Longo S. (2019). Water- and fat-soluble antioxidants in human seminal plasma and serum of fertile males. *Antioxidants* 8:96. 10.3390/antiox8040096 30978904 PMC6523754

[B100] Lee I. M. Cook N. R. Gaziano J. M. Gordon D. Ridker P. M. Manson J. E. (2005). Vitamin E in the primary prevention of cardiovascular disease and cancer: the Women’s Health Study: a randomized controlled trial. *Jama* 294 56–65. 15998891 10.1001/jama.294.1.56

[B101] Lee S. Q. Tan T. S. Kawamukai M. Chen E. S. (2017). Cellular factories for coenzyme Q10 production. *Microb. Cell Fact.* 16:39. 10.1186/s12934-017-0646-4 PMC5335738 28253886

[B102] Li H. Horke S. Forstermann U. (2014). Vascular oxidative stress, nitric oxide and atherosclerosis. *Atherosclerosis* 237 208–219. 10.1016/j.atherosclerosis.2014.09.001 25244505

[B103] Li J. Wuliji O. Li W. Jiang Z.-G. Ghanbari H. A. (2013). Oxidative stress and neurodegenerative disorders. *Int. J. Mol. Sci.* 14 24438–24475. 24351827 10.3390/ijms141224438 PMC3876121

[B104] Li W. Cao L. Han L. Xu Q. Ma Q. (2015). Superoxide dismutase promotes the epithelial-mesenchymal transition of pancreatic cancer cells via activation of the H2O2/ERK/NF-κB axis. *Int. J. Oncol.* 46 2613–2620. 10.3892/ijo.2015.2938 25825208

[B105] Liang X. Wang S. Wang L. Ceylan A. F. Ren J. Zhang Y. (2020). Mitophagy inhibitor liensinine suppresses doxorubicin-induced cardiotoxicity through inhibition of drp1-mediated maladaptive mitochondrial fission. *Pharmacol. Res.* 157:104846. 10.1016/j.phrs.2020.104846 32339784

[B106] Liguori I. Russo G. Aran L. Bulli G. Curcio F. Della-Morte D. (2018). Sarcopenia: assessment of disease burden and strategies to improve outcomes. *Clin. Interv. Aging* 13:913. 10.2147/cia.s149232 29785098 PMC5957062

[B107] Lin J. Cook N. R. Albert C. Zaharris E. Gaziano J. M. Van Denburgh M. (2009). Vitamins C and E and beta carotene supplementation and cancer risk: a randomized controlled trial. *J. Natl. Cancer Inst.* 101 14–23. 10.1093/jnci/djn438 19116389 PMC2615459

[B108] Liou G. Y. Doppler H. Delgiorno K. E. Zhang L. Leitges M. Crawford H. C. (2016). Mutant KRas-induced mitochondrial oxidative stress in acinar cells upregulates EGFR signaling to drive formation of pancreatic precancerous lesions. *Cell Rep.* 14 2325–2336. 10.1016/j.celrep.2016.02.029 26947075 PMC4794374

[B109] Liu Z.-Q. (2019). Bridging free radical chemistry with drug discovery: a promising way for finding novel drugs efficiently. *Eur. J. Med. Chem.* 189:112020. 10.1016/j.ejmech.2019.112020 32006794

[B110] Lü J.-M. Lin P. H. Yao Q. Chen C. (2010). Chemical and molecular mechanisms of antioxidants: experimental approaches and model systems. *J. Cell. Mol.Med.* 14 840–860. 10.1111/j.1582-4934.2009.00897.x 19754673 PMC2927345

[B111] Mach F. Schonbeck U. Libby P. (1998). CD40 signaling in vascular cells: a key role in atherosclerosis? *Atherosclerosis* 137(Suppl.), S89–S95. 9694547 10.1016/s0021-9150(97)00309-2

[B112] Mahajan L. Verma P. K. Raina R. Pankaj N. K. Sood S. Singh M. (2018). Alteration in thiols homeostasis, protein and lipid peroxidation in renal tissue following subacute oral exposure of imidacloprid and arsenic in Wistar rats. *Toxicol. Rep.* 5 1114–1119. 10.1016/j.toxrep.2018.11.003 30456172 PMC6231080

[B113] Marchitti S. A. Chen Y. Thompson D. C. Vasiliou V. (2011). Ultraviolet radiation: cellular antioxidant response and the role of ocular aldehyde dehydrogenase enzymes. *Eye Contact Lens* 37:206. 10.1097/icl.0b013e3182212642 21670692 PMC3356694

[B114] Marti R. Rosello S. Cebolla-Cornejo J. (2016). Tomato as a source of carotenoids and polyphenols targeted to cancer prevention. *Cancers* 8:58. 10.3390/cancers8060058 27331820 PMC4931623

[B115] Meybodi N. M. Mortazavian A. M. Monfared A. B. Sohrabvandi S. Meybodi F. A. (2017). Phytochemicals in Cancer prevention: a review of the evidence. *Int. J. Cancer Manag.* 10:e7219.

[B116] Miltonprabu S. Sumedha N. C. Senthilraja P. (2017). Diallyl trisulfide, a garlic polysulfide protects against As-induced renal oxidative nephrotoxicity, apoptosis and inflammation in rats by activating the Nrf2/ARE signaling pathway. *Int. Immunopharmacol.* 50 107–120. 10.1016/j.intimp.2017.06.011 28648972

[B117] Mishra A. P. Salehi B. Sharifi-Rad M. Pezzani R. Kobarfard F. Sharifi-Rad J. (2018). Programmed Cell death, from a cancer perspective: an overview. *Mol. Diagn. Ther.* 22 281–295. 29560608 10.1007/s40291-018-0329-9

[B118] Murr C. Schroecksnadel K. Winkler C. Ledochowski M. Fuchs D. (2005). Antioxidants may increase the probability of developing allergic diseases and asthma. *Med. Hypotheses* 64 973–977. 10.1016/j.mehy.2004.11.011 15780494

[B119] Niedzielska E. Smaga I. Gawlik M. Moniczewski A. Stankowicz P. Pera J. (2016). Oxidative stress in neurodegenerative diseases. *Mol. Neurobiol.* 53 4094–4125. 26198567 10.1007/s12035-015-9337-5 PMC4937091

[B120] Nussbaum L. Hogea L. M. Cãlina D. Andreescu N. Grãdinaru R.?tefãnescu R. (2017). Modern treatment approaches in psychoses. Pharmacogenetic, neuroimagistic and clinical implications. *Farmacia* 65 75–81.

[B121] Oke G. O. Abiodun A. A. Imafidon C. E. Monsi B. F. (2019). Zingiber officinale (Roscoe) mitigates CCl4-induced liver histopathology and biochemical derangements through antioxidant, membrane-stabilizing and tissue-regenerating potentials. *Toxicol. Rep.* 6 416–425. 10.1016/j.toxrep.2019.05.001 31193041 PMC6514439

[B122] Padureanu R. Albu C. V. Mititelu R. R. Bacanoiu M. V. Docea A. O. Calina D. (2019). Oxidative stress and inflammation interdependence in multiple sclerosis. *J. Clin. Med.* 8:1815. 10.3390/jcm8111815 31683787 PMC6912446

[B123] Panic N. Nedovic D. Pastorino R. Boccia S. Leoncini E. (2017). Carotenoid intake from natural sources and colorectal cancer: a systematic review and meta-analysis of epidemiological studies. *Eur. J. Cancer Prev.* 26 27–37. 10.1097/cej.0000000000000251 26960163

[B124] Papa S. Martino P. L. Capitanio G. Gaballo A. De Rasmo D. Signorile A. (2012). The oxidative phosphorylation system in mammalian mitochondria. *Adv. Exp. Med. Biol.* 942 3–37. 22399416 10.1007/978-94-007-2869-1_1

[B125] Park L. Zhou P. Pitstick R. Capone C. Anrather J. Norris E. H. (2008). Nox2-derived radicals contribute to neurovascular and behavioral dysfunction in mice overexpressing the amyloid precursor protein. *Proc. Natl. Acad. Sci. US.A.* 105 1347–1352. 10.1073/pnas.0711568105 18202172 PMC2234141

[B126] Park M. H. Jo M. Kim Y. R. Lee C. K. Hong J. T. (2016). Roles of peroxiredoxins in cancer, neurodegenerative diseases and inflammatory diseases. *Pharmacol. Ther.* 163 1–23. 10.1016/j.pharmthera.2016.03.018 27130805 PMC7112520

[B127] Pasinelli P. Belford M. E. Lennon N. Bacskai B. J. Hyman B. T. Trotti D. (2004). Amyotrophic lateral sclerosis-associated SOD1 mutant proteins bind and aggregate with Bcl-2 in spinal cord mitochondria. *Neuron* 43 19–30. 10.1016/j.neuron.2004.06.021 15233914

[B128] Payne B. A. I. Chinnery P. F. (2015). Mitochondrial dysfunction in aging: much progress but many unresolved questions. *Biochim. Biophys. Acta* 1847 1347–1353. 10.1016/j.bbabio.2015.05.022 26050973 PMC4580208

[B129] Peake J. Suzuki K. (2004). Neutrophil activation, antioxidant supplements and exercise-induced oxidative stress. *Exerc. Immunol. Rev.* 10 129–141. 15633591

[B130] Pei Z. Deng Q. Babcock S. A. He E. Y. Ren J. Zhang Y. (2018). Inhibition of advanced glycation endproduct (AGE) rescues against streptozotocin-induced diabetic cardiomyopathy: role of autophagy and ER stress. *Toxicol. Lett.* 284 10–20. 10.1016/j.toxlet.2017.11.018 29174818

[B131] Perera N. C. N. Godahewa G. I. Lee S. Kim M. J. Hwang J. Y. Kwon M. G. (2017). Manganese-superoxide dismutase (MnSOD), a role player in seahorse (*Hippocampus abdominalis*) antioxidant defense system and adaptive immune system. *Fish Shellfish Immunol.* 68 435–442. 10.1016/j.fsi.2017.07.049 28743628

[B132] Perrotta I. Aquila S. (2015). The role of oxidative stress and autophagy in atherosclerosis. *Oxid. Med. Cell. Longev.* 2015:130315. 10.1155/2015/130315 PMC4381688 25866599

[B133] Pingitore A. Lima G. P. P. Mastorci F. Quinones A. Iervasi G. Vassalle C. (2015). Exercise and oxidative stress: potential effects of antioxidant dietary strategies in sports. *Nutrition* 31 916–922. 10.1016/j.nut.2015.02.005 26059364

[B134] Pizzino G. Bitto A. Interdonato M. Galfo F. Irrera N. Mecchio A. (2014). Oxidative stress and DNA repair and detoxification gene expression in adolescents exposed to heavy metals living in the Milazzo-Valle del Mela area (Sicily. Italy). *Redox. Biol.* 2 686–693. 10.1016/j.redox.2014.05.003 24936443 PMC4052524

[B135] Pizzino G. Irrera N. Cucinotta M. Pallio G. Mannino F. Arcoraci V. (2017). Oxidative stress: harms and benefits for human health. *Oxid. Med. Cell. Longev.* 2017:8416763. 10.1155/2017/8416763 PMC5551541 28819546

[B136] Poli G. Leonarduzzi G. Biasi F. Chiarpotto E. (2004). Oxidative stress and cell signalling. *Curr. Med. Chem.* 11 1163–1182. 10.2174/0929867043365323 15134513

[B137] Poole L. B. Nelson K. J. (2016). Distribution and features of the six classes of peroxiredoxins. *Mol. Cells* 39 53–59. 10.14348/molcells.2016.2330 26810075 PMC4749874

[B138] Poprac P. Jomova K. Simunkova M. Kollar V. Rhodes C. J. Valko M. (2017). Targeting free radicals in oxidative stress-related human diseases. *Trends Pharmacol. Sci.* 38 592–607. 10.1016/j.tips.2017.04.005 28551354

[B139] Prochazkova D. Bousova I. Wilhelmova N. (2011). Antioxidant and prooxidant properties of flavonoids. *Fitoterapia* 82 513–523. 10.1016/j.fitote.2011.01.018 21277359

[B140] Ramsay R. R. (2019). Electron carriers and energy conservation in mitochondrial respiration. *Chem. Texts* 5:9.

[B141] Rasouli H. Farzaei M. H. Mansouri K. Mohammadzadeh S. Khodarahmi R. (2016). Plant cell cancer: may natural phenolic compounds prevent onset and development of plant cell malignancy? A literature review. *Molecules* 21:1104. 10.3390/molecules21091104 27563858 PMC6274315

[B142] Ray P. D. Huang B.-W. Tsuji Y. (2012). Reactive oxygen species (ROS) homeostasis and redox regulation in cellular signaling. *Cell. Signal.* 24 981–990. 10.1016/j.cellsig.2012.01.008 22286106 PMC3454471

[B143] Reddy P. H. (2009). Role of mitochondria in neurodegenerative diseases: mitochondria as a therapeutic target in Alzheimer’s disease. *CNS Spectr.* 14 8–18. 10.1017/s1092852900024901 PMC3056539 19890241

[B144] Reid M. B. (2001). Invited Review: redox modulation of skeletal muscle contraction: what we know and what we don’t. *J. Appl. Physiol.* 90 724–731. 10.1152/jappl.2001.90.2.724 11160074

[B145] Ren J. Taegtmeyer H. (2015). Too much or not enough of a good thing—The Janus faces of autophagy in cardiac fuel and protein homeostasis. *J. Mol. Cell. Cardiol.* 84 223–226. 10.1016/j.yjmcc.2015.03.001 25771142

[B146] Reuter S. Gupta S. C. Chaturvedi M. M. Aggarwal B. B. (2010). Oxidative stress, inflammation, and cancer: how are they linked? *Free Radic. Biol. Med.* 49 1603–1616. 10.1016/j.freeradbiomed.2010.09.006 20840865 PMC2990475

[B147] Riederer P. Sofic E. Rausch W. D. Schmidt B. Reynolds G. P. Jellinger K. (1989). Transition metals, ferritin, glutathione, and ascorbic acid in parkinsonian brains. *J. Neurochem.* 52 515–520. 10.1111/j.1471-4159.1989.tb09150.x 2911028

[B148] Rodriguez R. Redman R. (2005). Balancing the generation and elimination of reactive oxygen species. *Proc. Natl. Acad. Sci.* 102 3175–3176. 10.1073/pnas.0500367102 15728396 PMC552941

[B149] Roychoudhury S. Agarwal A. Virk G. Cho C. L. (2017). Potential role of green tea catechins in the management of oxidative stress-associated infertility. *Reprod. Biomed. Online* 34 487–498. 10.1016/j.rbmo.2017.02.006 28285951

[B150] Saccon R. A. Bunton-Stasyshyn R. K. Fisher E. M. Fratta P. (2013). Is SOD1 loss of function involved in amyotrophic lateral sclerosis? *Brain* 136 2342–2358. 10.1093/brain/awt097 23687121 PMC3722346

[B151] Sackesen C. Ercan H. Dizdar E. Soyer O. Gumus P. Tosun B. N. (2008). A comprehensive evaluation of the enzymatic and nonenzymatic antioxidant systems in childhood asthma. *J. Allergy Clin. Immunol.* 122 78–85. 10.1016/j.jaci.2008.03.035 18485467

[B152] Saez I. Vilchez D. (2014). The mechanistic links between proteasome activity, aging and age-related diseases. *Curr. Genomics* 15 38–51. 10.2174/138920291501140306113344 24653662 PMC3958958

[B153] Sage E. Girard P.-M. Francesconi S. (2012). Unravelling UVA-induced mutagenesis. *Photochem. Photobiol. Sci.* 11 74–80. 10.1039/c1pp05219e 21901217

[B154] Saha S. K. Lee S. B. Won J. Choi H. Y. Kim K. Yang G. M. (2017). Correlation between oxidative stress, nutrition, and cancer initiation. *Int. J. Mol. Sci.* 18:1544. 10.3390/ijms18071544 28714931 PMC5536032

[B155] Salehi B. Calina D. Docea A. O. Koirala N. Aryal S. Lombardo D. (2020a). Curcumin’s nanomedicine formulations for therapeutic application in neurological diseases. *J. Clin. Med.* 9:430. 10.3390/jcm9020430 32033365 PMC7074182

[B156] Salehi B. Rescigno A. Dettori T. Calina D. Docea A. O. Singh L. (2020b). Avocado–soybean unsaponifiables: a panoply of potentialities to be exploited. *Biomolecules* 10:130. 10.3390/biom10010130 31940989 PMC7023362

[B157] Salehi B. Capanoglu E. Adrar N. Catalkaya G. Shaheen S. Jaffer M. (2019a). *Cucurbits* plants: a key emphasis to its pharmacological potential. *Molecules* 24:1854. 10.3390/molecules24101854 31091784 PMC6572650

[B158] Salehi B. Lopez-Jornet P. Pons-Fuster López E. Calina D. Sharifi-Rad M. Ramírez-Alarcón K. (2019b). Plant-derived bioactives in oral mucosal lesions: a key emphasis to curcumin, lycopene, chamomile, aloe vera, green tea and coffee properties. *Biomolecules* 9:106. 10.3390/biom9030106 30884918 PMC6468600

[B159] Salehi B. Sestito S. Rapposelli S. Peron G. Calina D. Sharifi-Rad M. (2019c). Epibatidine: a promising natural alkaloid in health. *Biomolecules* 9:6. 10.3390/biom9010006 30583611 PMC6359223

[B160] Salehi B. Shivaprasad Shetty M. V. Anil Kumar N. Živkoviæ J. Calina D. Oana Docea A. (2019d). *Veronica* Plants—Drifting from farm to traditional healing, food application, and phytopharmacology. *Molecules* 24:2454. 10.3390/molecules24132454 31277407 PMC6651156

[B161] Salehi B. Martorell M. Arbiser J. L. Sureda A. Martins N. Maurya P. K. (2018). Antioxidants: positive or negative actors? *Biomolecules* 8:124. 10.3390/biom8040124 30366441 PMC6316255

[B162] Sani T. A. Mohammadpour E. Mohammadi A. Memariani T. Yazdi M. V. Rezaee R. (2017). Cytotoxic and apoptogenic properties of *Dracocephalum kotschyi* aerial part different fractions on calu-6 and mehr-80 lung cancer cell lines. *Farmacia* 65 189–199.

[B163] Sanjust E. Mocci G. Zucca P. Rescigno A. (2008). Mediterranean shrubs as potential antioxidant sources. *Nat. Prod. Res.* 22 689–708. 10.1080/14786410801997125 18569710

[B164] Schottker B. Brenner H. Jansen E. H. Gardiner J. Peasey A. Kubinova R. (2015). Evidence for the free radical/oxidative stress theory of ageing from the *CHANCES consortium*: a meta-analysis of individual participant data. *BMC Med.* 13:300. 10.1186/s12916-015-0537-7 26666526 PMC4678534

[B165] Ściskalska M. Zalewska M. Grzelak A. Milnerowicz H. (2014). The influence of the occupational exposure to heavy metals and tobacco smoke on the selected oxidative stress markers in smelters. *Biol. Trace Element Res.* 159 59–68. 10.1007/s12011-014-9984-9 24789476 PMC4051999

[B166] Sena L. A. Chandel N. S. (2012). Physiological roles of mitochondrial reactive oxygen species. *Mol. Cell* 48 158–167. 10.1016/j.molcel.2012.09.025 23102266 PMC3484374

[B167] Seo M. Y. Lee S. M. (2002). Protective effect of low dose of ascorbic acid on hepatobiliary function in hepatic ischemia/reperfusion in rats. *J. Hepatol.* 36 72–77. 10.1016/s0168-8278(01)00236-7 11804667

[B168] Sharifi-Rad J. Rodrigues C. F. Sharopov F. Docea A. O. Can Karaca A. Sharifi-Rad M. (2020). Diet, lifestyle and cardiovascular diseases: linking pathophysiology to cardioprotective effects of natural bioactive compounds. *Int. J. Environ. Res. Public Health* 17:2326. 10.3390/ijerph17072326 32235611 PMC7177934

[B169] Sharifi-Rad M. Lankatillake C. Dias D. A. Docea A. O. Mahomoodally M. F. Lobine D. (2020). Impact of natural compounds on neurodegenerative disorders: from preclinical to pharmacotherapeutics. *J. Clin. Med.* 9:1061. 10.3390/jcm9041061 32276438 PMC7231062

[B170] Sharifi-Rad J. Sharifi-Rad M. Salehi B. Iriti M. Roointan A. Mnayer D. (2018). In vitro and in vivo assessment of free radical scavenging and antioxidant activities of *Veronica persica* Poir. *Cell. Mol. Biol.* 64 57–64. 29981684

[B171] Sharma P. Jha A. B. Dubey R. S. Pessarakli M. (2012). reactive oxygen species, oxidative damage, and antioxidative defense mechanism in plants under stressful conditions. *J. Bot.* 2012:26.

[B172] Singh R. B. Mengi S. A. Xu Y. J. Arneja A. S. Dhalla N. S. (2002). Pathogenesis of atherosclerosis: a multifactorial process. *Exp. Clin. Cardiol.* 7 40–53. 19644578 PMC2716189

[B173] Singh Y. P. Patel R. N. Singh Y. Butcher R. J. Vishakarma P. K. Singh R. K. B. (2017). Structure and antioxidant superoxide dismutase activity of copper(II) hydrazone complexes. *Polyhedron* 122 1–15. 10.1016/j.poly.2016.11.013

[B174] Smith M. T. Guyton K. Z. Gibbons C. F. Fritz J. M. Portier C. J. Rusyn I. (2016). Key characteristics of carcinogens as a basis for organizing data on mechanisms of carcinogenesis. *Environ. Health Perspect.* 124 713–721. 10.1289/ehp.1509912 26600562 PMC4892922

[B175] Sova H. Jukkola-Vuorinen A. Puistola U. Kauppila S. Karihtala P. (2010). 8-Hydroxydeoxyguanosine: a new potential independent prognostic factor in breast cancer. *Br. J. Cancer* 102 1018–1023. 10.1038/sj.bjc.6605565 20179711 PMC2844025

[B176] Spitz D. R. Azzam E. I. Li J. J. Gius D. (2004). Metabolic oxidation/reduction reactions and cellular responses to ionizing radiation: a unifying concept in stress response biology. *Cancer Metast. Rev.* 23 311–322. 10.1023/b:canc.0000031769.14728.bc 15197331

[B177] Spitz D. R. Hauer-Jensen M. (2014). *Ionizing Radiation-Induced Responses: Where Free Radical Chemistry Meets Redox Biology and Medicine.* Rochelle, NY: Mary Ann Liebert, Inc. 10.1089/ars.2013.5769 PMC3936498 24354361

[B178] Tafazoli A. (2017). Coenzyme Q10 in breast cancer care. *Future Oncol.* 13 1035–1041. 10.2217/fon-2016-0547 28481148

[B179] Takalo M. Salminen A. Soininen H. Hiltunen M. Haapasalo A. (2013). Protein aggregation and degradation mechanisms in neurodegenerative diseases. *Am. J. Neurodegenerat. Dis.* 2 1–14. PMC3601466 23516262

[B180] Taverne Y. J. Bogers A. J. Duncker D. J. Merkus D. (2013). Reactive oxygen species and the cardiovascular system. *Oxid. Med. Cell. Longev.* 2013:862423. 10.1155/2013/862423 PMC3655680 23738043

[B181] Tovmasyan A. Maia C. G. Weitner T. Carballal S. Sampaio R. S. Lieb D. (2015). A comprehensive evaluation of catalase-like activity of different classes of redox-active therapeutics. *Free Radic. Biol. Med.* 86 308–321. 10.1016/j.freeradbiomed.2015.05.018 26026699 PMC4554972

[B182] Tsatsakis A. Docea A. O. Calina D. Tsarouhas K. Zamfira L.-M. Mitrut R. (2019). A mechanistic and pathophysiological approach for stroke associated with drugs of abuse. *J. Clin. Med.* 8:1295. 10.3390/jcm8091295 31450861 PMC6780697

[B183] Tsatsakis A. M. Docea A. O. Calina D. Buga A. M. Zlatian O. Gutnikov S. (2019). Hormetic Neurobehavioral effects of low dose toxic chemical mixtures in real-life risk simulation (RLRS) in rats. *Food Chem. Toxicol.* 125 141–149. 10.1016/j.fct.2018.12.043 30594548

[B184] Tse G. Yan B. P. Chan Y. W. Tian X. Y. Huang Y. (2016). Reactive oxygen species, endoplasmic reticulum stress and mitochondrial dysfunction: the link with cardiac arrhythmogenesis. *Front. Physiol.* 7:313. 10.3389/fphys.2016.00313 27536244 PMC4971160

[B185] Tsoukalas D. Fragkiadaki P. Docea A. O. Alegakis A. K. Sarandi E. Thanasoula M. (2019a). Discovery of potent telomerase activators: unfolding new therapeutic and anti-aging perspectives. *Mol. Med. Rep.* 20 3701–3708. 31485647 10.3892/mmr.2019.10614 PMC6755196

[B186] Tsoukalas D. Fragkiadaki P. Docea A. O. Alegakis A. K. Sarandi E. Vakonaki E. (2019b). Association of nutraceutical supplements with longer telomere length. *Int. J. Mol. Med.* 44 218–226. 31115552 10.3892/ijmm.2019.4191 PMC6559326

[B187] Tsoukalas D. Fragoulakis V. Sarandi E. Docea A. O. Papakonstantinou E. Tsilimidos G. (2019c). Targeted metabolomic analysis of serum fatty acids for the prediction of autoimmune diseases. *Front. Mol. Biosci.* 6:120. 10.3389/fmolb.2019.00120 31737644 PMC6839420

[B188] Valavanidis A. Vlachogianni T. Fiotakis K. (2009). Tobacco smoke: involvement of reactive oxygen species and stable free radicals in mechanisms of oxidative damage, carcinogenesis and synergistic effects with other respirable particles. *Int. J. Environ. Res. Public Health* 6 445–462. 10.3390/ijerph6020445 19440393 PMC2672368

[B189] Valko M. Leibfritz D. Moncol J. Cronin M. T. Mazur M. Telser J. (2007). Free radicals and antioxidants in normal physiological functions and human disease. *Int. J. Biochem. Cell Biol.* 39 44–84. 10.1016/j.biocel.2006.07.001 16978905

[B190] Valko M. Rhodes C. J. Moncol J. Izakovic M. Mazur M. (2006). Free radicals, metals and antioxidants in oxidative stress-induced cancer. *Chem. Biol. Interact.* 160 1–40. 10.1016/j.cbi.2005.12.009 16430879

[B191] Wang X. Michaelis E. K. (2010). Selective neuronal vulnerability to oxidative stress in the brain. *Front. Aging Neurosci.* 2:12. 10.3389/fnagi.2010.00012 20552050 PMC2874397

[B192] Watson R. R. (1998). *Melatonin in the Promotion of Health*, 2nd Edn Boca Raton, FL: Taylor & Francis Group.

[B193] Wattanapitayakul S. K. Bauer J. A. (2001). Oxidative pathways in cardiovascular disease: roles, mechanisms, and therapeutic implications. *Pharmacol. Ther.* 89 187–206. 10.1016/s0163-7258(00)00114-5 11316520

[B194] Wu N. N. Tian H. Chen P. Wang D. Ren J. Zhang Y. (2019). Physical exercise and selective autophagy: benefit and risk on cardiovascular health. *Cell* 8:1436. 10.3390/cells8111436 31739509 PMC6912418

[B195] Wu X. Liu X. Huang H. Li Z. Xiong T. Xiang W. (2019). Effects of major ozonated autoheamotherapy on functional recovery, ischemic brain tissue apoptosis and oxygen free radical damage in the rat model of cerebral ischemia. *J. Cell. Biochem.* 120 6772–6780. 10.1002/jcb.27978 30390335

[B196] Ye G. Metreveli N. S. Donthi R. V. Xia S. Xu M. Carlson E. C. (2004). Catalase protects cardiomyocyte function in models of type 1 and type 2 diabetes. *Diabetes* 53 1336–1343. 10.2337/diabetes.53.5.1336 15111504

[B197] Young A. J. Lowe G. M. (2001). Antioxidant and prooxidant properties of carotenoids. *Arch. Biochem. Biophys.* 385 20–27. 10.1006/abbi.2000.2149 11361018

[B198] Zal F. Taheri R. Khademi F. Keshavarz E. Rajabi S. Mostafavi-Pour Z. (2014). The combined effect of furosemide and propranolol on GSH homeostasis in ACHN renal cells. *Toxicol. Mech. Methods* 24 412–416. 10.3109/15376516.2014.926437 24845846

[B199] Zhang J. McCullough P. A. (2016). Lipoic acid in the prevention of acute kidney injury. *Nephron* 134 133–140. 10.1159/000448666 27603173

[B200] Zhang W. Wang T. Qin L. Gao H. M. Wilson B. Ali S. F. (2004). Neuroprotective effect of dextromethorphan in the MPTP Parkinson’s disease model: role of NADPH oxidase. *Faseb. J.* 18 589–591. 10.1096/fj.03-0983fje 14734632

[B201] Zhou H. Wang S. Zhu P. Hu S. Chen Y. Ren J. (2018). Empagliflozin rescues diabetic myocardial microvascular injury via AMPK-mediated inhibition of mitochondrial fission. *Redox Biol.* 15 335–346. 10.1016/j.redox.2017.12.019 29306791 PMC5756062

[B202] Zhu X. Jiang S. Hu N. Luo F. Dong H. Kang Y. M. (2014). Tumour necrosis factor-α inhibition with lenalidomide alleviates tissue oxidative injury and apoptosis in ob/ob obese mice. *Clin. Exp. Pharmacol. Physiol.* 41 489–501. 10.1111/1440-1681.12240 24739012

[B203] Zucca P. Argiolas A. Nieddu M. Pintus M. Rosa A. Sanna F. (2016). Biological activities and nutraceutical potentials of water extracts from different parts of cynomorium coccineum L. (*Maltese Mushroom*). *Polish J. Food Nutr.l Sci.* 66 179–188. 10.1515/pjfns-2016-0006

[B204] Zucca P. Rescigno A. Rinaldi A. C. Sanjust E. (2014). Biomimetic metalloporphines and metalloporphyrins as potential tools for delignification: molecular mechanisms and application perspectives. *J. Mol. Catal. A Chem.* 388-389 2–34. 10.1016/j.molcata.2013.09.010

